# Enhancing sports image data classification in federated learning through genetic algorithm-based optimization of base architecture

**DOI:** 10.1371/journal.pone.0303462

**Published:** 2024-07-11

**Authors:** De Sheng Fu, Jie Huang, Dibyanarayan Hazra, Amit Kumar Dwivedi, Suneet Kumar Gupta, Basu Dev Shivahare, Deepak Garg

**Affiliations:** 1 College of Public Education, ZheJiang Institute of Economics and Trade HangZhou, ZheJiang, China; 2 College of Business administration ZheJiang Institute of Economics and Trade HangZhou, ZheJiang, China; 3 School of Computer Science Engineering and Technology, Bennett University, Greater Noida, India; 4 School of Computer Science, UPES, Dehradun, India; 5 Galgotias University, Gr. Noida, India; 6 SR University, Warangal, India; Firat Universitesi, TURKEY

## Abstract

Nowadays, federated learning is one of the most prominent choices for making decisions. A significant benefit of federated learning is that, unlike deep learning, it is not necessary to share data samples with the model owner. The weight of the global model in traditional federated learning is created by averaging the weights of all clients or sites. In the proposed work, a novel method has been discussed to generate an optimized base model without hampering its performance, which is based on a genetic algorithm. Chromosome representation, crossover, and mutation—all the intermediate operations of the genetic algorithm have been illustrated with useful examples. After applying the genetic algorithm, there is a significant improvement in inference time and a huge reduction in storage space. Therefore, the model can be easily deployed on resource-constrained devices. For the experimental work, sports data has been used in balanced and unbalanced scenarios with various numbers of clients in a federated learning environment. In addition, we have used four famous deep learning architectures, such as AlexNet, VGG19, ResNet50, and EfficientNetB3, as the base model. We have achieved 92.34% accuracy with 9 clients in the balanced data set by using EfficientNetB3 as the base model using a GA-based approach. Moreover, after applying the genetic algorithm to optimize EfficientNetB3, there is an improvement in inference time and storage space by 20% and 2.35%, respectively.

## 1. Introduction & related work

Artificial intelligence (AI) has received a huge interest nowadays because of numerous applications in the fields of healthcare, education, security monitoring, and agriculture [1–5]. In AI, computer systems learn from the given data and statistical patterns to predict an accurate result based on the extracted knowledge using AI techniques [6, 7].

Machine learning (ML) algorithms come in a variety of flavors, including supervised learning, unsupervised learning, and reinforcement learning. When employing supervised learning (SL), each input data point receives the proper response or output since the system is trained using labeled data [8]. Unsupervised learning teaches the system using unlabeled data, so the results are initially ambiguous. When a system receives feedback in the form of rewards or penalties depending on its conduct, it learns through reinforcement [9].

Deep learning (DL), on the other hand, can handle more challenging problems and replicate the process of human learning. Another significant benefit of DL over ML is that less feature engineering is needed because DL models automatically learn features from input images [10]. Additionally, DL is more accurate than ML models, but DL has a few drawbacks, such as the complicated model’s need for a lot of space and powerful computation during the system’s training. However, since the DL model needed a lot of data to be trained, collecting that data became the major obstacle to applying DL models in practical applications [11]. It is established that DL is an effective technique for handling complicated decision-making problems, but there are still certain concerns, including those related to data privacy, infrastructure, communication costs, etc. [12]. However, Federated Learning (FL) can solve these obstacles in deep learning.

Federated learning is a machine learning technique that allows several parties to work together to train a single model while maintaining the privacy and decentralization of their own data [13]. In FL, a model is shared and trained utilizing information from several sources that have access to information of a similar nature. Each site shares model-related data with a centralized server once the model has been trained across all sites, and the server then averages the weights to create the aggregated model. This process must be done several times until the optimal global model is not found [14, 15].

Sports are becoming a crucial component of both international trade and leisure. Athletic ability is important in sports. The study’s authors gathered player performance feature vectors and summaries of game statistics. They then used k-fold cross-validation to test the feature vectors and the Genetic Algorithm (GA) to combine the best feature subsets.

Chan et al. [16] described where to find particular classifications of ice hockey players, such as defenders, strikers, etc. The authors used the clustering method. They were able to establish a connection between the various player types clustered together and the team’s success using a regression model for these clusters. Team management can use the Excel-based tool the writers offered to assess new contracts and the addition of new players. Ahmed et al. [17] outlined a method for assembling a world-class cricket team that uses the least amount of resources and the maximum performance.

In [18], Based on the surroundings, the authors have given a strong foundation for classifying sports images. The authors also asserted that their approach relies on the use of Inception V3 for feature extraction and neural networks for sports classification. Six sports have been used for analysis and categorization. HAR places a particular emphasis on sports. In [19], The European handball data set, which can be divided into six different sports groups, is analyzed using the provided motion descriptors and SVM classification in the authors’ technique to detect team actions. The Poisson equation was employed in this manner to generate a smooth distribution that encompassed the entire playground because the team members’ exact placements on the ground were known. Additionally, position distribution was used to refer to smooth distribution.

In [20], authors have studied the process of gathering body area sensors for sports identification. Additionally, sensors are installed in the player’s body parts, like their legs and arms, and the information they acquire is kept in one location.

A summary of the study conducted by the researchers in the same field is shown in the following Table 1.

**Table 1:** Literature review summary.

Author	Title	Description	Highlights
Ketan Joshi et al. [18]	Robust sports image classification using inceptionV3 and neural networks	It presents a framework for sports image classification using Inception V3 and neural network and achieves an average accuracy of 96.64% over six sports categories. A detailed comparison is also given with other classifiers such as Random Forest, K-Nearest Neighbors, etc., for effectiveness validation.	In this work, sports images and videos are analyzed to develop various applications such as blog writing, sports education, etc.
Russo et al. [21]	Classification of sports videos with combination of deep learning models and transfer learning	It proposes a deep learning-based approach that combines convolutional and recurrent neural networks to classify sports videos into 15 individual classes, achieving high test accuracy using transfer learning with the VGG-16 model.	The proposed approach focuses on sports action-based classification by combining spatial and motion features extracted from CNN with temporal analysis using RNN.
SSkandha et al. [22]	A novel genetic algorithm-based approach for compression and acceleration of deep learning convolution neural network: an application in computer tomography lung cancer data	This work highlights the compression of deep neural network for ensuring its suitability towards IOT devices. Since lung cancer is one of a life-threatening diseases, it is essential to be detected using low-configuration devices at an early stage. This work uses a Genetic Algorithm for model compression where the unwanted layers in the neural network are removed to improve the efficiency of the model.	The proposed approach reduces 90.3% storage space and also improves the inference time by 35%.
Petrini et al. [23]	Deep neural networks compression: A comparative survey and choice recommendations	The paper presents a comprehensive comparison of lossy and structure-preserving approaches to compress pre-trained convolutional neural networks (CNNs) and provides guidance for choosing the most suitable compression technique. The study includes experiments on two state-of-the-art CNNs and five benchmarks, analyzing the performance of compression techniques on both convolutional and fully-connected layers for classification and regression problems.	The experimental setting used to compare the compression techniques and described, including the use of two pre-trained CNN models and five datasets.
Simon Wiedemann et al. [24]	DeepCABAC: A Universal Compression Algorithm for Deep Neural Networks	DeepCABAC is a compression algorithm for deep neural networks (DNNs) that applies Context-based Adaptive Binary Arithmetic Coder (CABAC) to the DNN parameters, achieving higher compression rates than previous techniques for DNN compression. It uses a novel quantization scheme that minimizes a rate-distortion function while considering the impact of quantization on DNN performance, allowing the representation of the entire network with just 9 MB.	The algorithm is based on the H.264/AVC video coding standard and applies CABAC, which is a state-of-the-art lossless compression technique for video compression. Experimental results show that DeepCABAC consistently achieves higher compression rates compared to previously proposed coding techniques for DNN compression.
Podgorelec, V. et al. [25]	Classification of similar sports images using a convolutional neural network with hyper-parameter optimization	The paper discusses the use of transfer learning in image classification, specifically for classifying sports images. The paper discusses the use of transfer learning in image classification, specifically for classifying sports images. It presents a proposed image classification method and describes the conducted experiments and results. The authors also discuss the interpretation of the trained models using methods like LIME and SHAP.	The paper explores the use of transfer learning techniques, specifically fine-tuning, for image classification. Transfer learning involves training a model on a pre-trained model with adapted weight values, reducing training time and potentially improving predictive performance.
Gao, Y. et al. [26]	Improved spatial pyramid matching for sports image classification	The paper addresses the need to consider both human pose and event scenes in sports image classification, using a combination of spatial pyramid matching (SPM) and Visual Words Spatial Dependence Matrices to improve classification accuracy. Experimental results show that the proposed method improves classification accuracy by approximately 19% compared to SPM and outperforms other improved SPM methods in sports image classification.	The paper also mentions the use of the KSPM method, which focuses on improving the spatial position of objectives in sports images. It shows effective improvement in classification accuracy, particularly for sports scenes with athletes on a large scale.
Huang, Pu. [27]	Sports Image Classification and Application Based on Visual Attention Analysis	The paper focuses on the classification and application of sports images using visual attention analysis, which simulates human eye recognition patterns and improves accuracy in classifying sports pictures. The study establishes a sports image classification system based on visual attention analysis and compares its effectiveness with other methods, showing significant advantages in terms of accuracy.	The results of the experiments show that the proposed method achieves an average accuracy of 34.5%, which is significantly higher than the visual impairment method (8.5%) and the core technology method (11.2%).
Sarma, Moumita Sen, et al. [28]	Traditional Bangladeshi sports video classification using deep learning method	The paper focuses on the classification of traditional Bangladeshi sports videos using deep learning techniques, specifically convolutional neural network (CNN) and long short term memory (LSTM) algorithms. A new dataset called Traditional Bangladeshi Sports Video (TBSV) is constructed, containing five classes of sports. The proposed model, which combines CNN and LSTM, outperforms previous works on challenging datasets and achieves an average accuracy of 99% on the TBSV dataset.	The spatial features of sequential frames are extracted using CNN and then fed to an LSTM layer for analysis.
Campr, Pavel, et al. [29]	Sports video classification in continuous TV broadcasts	The paper focuses on classifying video footage or continuous TV broadcasts based on their content, using categories such as talk show, sport, movie, cartoon, and more specific topics like summer and winter Olympic sports. The classification is done by analyzing each frame of the video separately and then filtering the results in the time domain for more accurate and robust classification. The paper also discusses the selection of robust image features and classifiers, showing that complex features based on convolutional neural networks outperform simple feature extractors.	The paper compares several feature extraction methods and classifiers for image scene classification and topic classification in continuous videos. It applies these methods to standalone images as well as continuous videos without prior knowledge of topic changes. Cross-validation is used for more robust results, and the experiments are repeated with three random splits of the data.
Farhad, Mohammad Yasir, et al. [30]	Sports-net18: Various sports classification using transfer learning	The paper proposes a VGG16 transfer learning model to classify eighteen categories of various sports, achieving a promising result of 93% accuracy. The authors have created their own sports dataset containing 9000 images and used deep learning techniques to accurately recognize and classify objects from sports images.	The proposed system consists of five convolutional blocks with different filter sizes and activation functions, followed by max-pooling and flattening layers. The authors have created their own sports dataset containing 9000 images for training and evaluation purposes.
Song, H. [31]	Secure prediction and assessment of sports injuries using deep learning based convolutional neural network	The paper discusses the use of an optimized convolutional neural network (OCNN) based on deep learning to detect and assess sports injuries. It focuses on the extraction, study, and accuracy of complex algorithms for analyzing sports medical data. The OCNN model includes two convolutional layers, two pool layers, a fully connected layer, and a SoftMax structure for classification. The paper also proposes a cloud-based loop model for creating an advanced medical data network for sports medicine. Experimental results show that this approach provides technical support and guidance for deploying a specific cloud-based fusion system.	The OCNN algorithm is used for data processing in the in-loop fusion simulation model, where the collected data is passed through the control layer and sent to the stored data center for processing. The paper suggests the use of a self-coding convolution neural network (SC neural network) that incorporates the configuration of the neural network of self-coding convolution to process and analyze multidimensional data.

In the proposed article, federated learning has been used for the classification of sports, with the generation of a global model by averaging the weights. In addition, we have also developed a method based on a Genetic Algorithm (GA) to obtain an optimized base model to improve the inference time and reduce of storage space of the trained model so that it can be easily deployed on resource-constrained devices. Our major contributions to the proposed study are as follows:

- Use of federated learning with a varying number of clients for the classification of unbalanced or balanced sports data with an unbalanced distribution over clients. Moreover, the global weight-averaging method has been used for the development of a generalized model to maintain data privacy.
- Developed a novel method to find the optimized base model for FL using a genetic algorithm.
- Design of a novel fitness function to check the strength of chromosomes. To develop the fitness function, three parameters have been used. 1) average accuracy 2) average loss in the federated learning model, and 3) number of hidden units in the optimized structure.
- A lot of tests have been done with well-known deep learning architectures like AlexNet, ResNet50, VGG19, and EfficientNetB3 by changing the number of clients on both balanced and unbalanced sports datasets.
- The experiment’s goal is to see how effective the global average strategy is at reducing storage while minimizing the inference time after applying the genetic algorithm to minimize the hidden units in the base architecture.

The structure of the article is as follows: A discussion about the data set used in the study is discussed in Section 2. A discussion about used terminologies and problem formulation is presented in Section 3. Introduction to federated learning, federated learning model generation using global averaging, and generation of an optimal base model for FL are discussed in Section 4. The experimental setup and result discussion are presented in Section 5. The conclusion is presented in section 6.

## 2 Dataset

A dataset is essential to perform a test for any machine or deep learning model. There are several datasets of sports available over the internet, but for this article, we have selected the dataset, which consists of 16 classes of different sports with different numbers of images in each class [32–34]. This data set is unbalanced, and we have applied different augmentation techniques, such as zoom-in, zoom-out, rotation, varying the light intensity, etc., to make the dataset balanced. In this article, we have tested our model in both unbalanced and balanced datasets. In the Table 2, it is shown the number of images per class before and after augmentation. We have divided the dataset into train, validation, and test sets for experimental work.

**Table 2:** Samples in each sport category in unbalanced and balanced condition.

Sport category	Number of samples					
	Unbalanced dataset			Balanced dataset		
	Training	Validation	Testing	Training	Validation	Testing
Cricket	413	118	63	650	270	100
Badminton	579	168	84	650	270	100
Football	459	130	64	650	270	100
Tennis	420	125	63	650	270	100
Basketball	285	78	38	650	270	100
Boxing	431	125	61	650	270	100
Chess	293	86	43	650	270	100
Formulaone	420	121	64	650	270	100
Gymnastics	393	121	63	650	270	100
Hockey	329	106	54	650	270	100
Kabaddi	281	77	42	650	270	100
Motogp	441	119	66	650	270	100
Shooting	335	91	49	650	270	100
Swimming	434	121	60	650	270	100
Volleyball	444	129	64	650	270	100
Wrestling	327	94	48	650	270	100

## 3 Terminologies and problem formulation

In the proposed work, we have used famous architectures as a base model in the federated learning environment for the classification of sports and after experiments, the best architecture has been selected for optimization purposes. A genetic algorithm has been used for the optimization of the model. In this section, first, a discussion about terminologies is presented, and based on the terminologies problem formulation has been taken place.

### 3.1 Terminologies

The following terminologies have been used in the proposed work.

- *N* represents the number of clients in the federated learning environment, and 1≤ *N* ≤10.
- *ρ*_*i*_ denotes the *i*^*th*^ base model of federated learning.
- *π*_*i*_ denotes the loss of the *i*^*th*^ base model in federated learning. In our proposed work, we have used the categorical cross-entropy loss function, which is represented mathematically in Eq 1.
  $$\begin{array}{c} \text{loss}\;\text{(x,}\;\text{a)}=-\sum_{p=1}^{m}x_{p}*log\left(a_{p}\right) \end{array}$$ (1)
  *x* represents the original probability distribution and *a* represents the predicted probability distribution. *p* denotes the number of classes in the classification problem.
-
$N=\{N_{1},N_{2}$ , $N_{3}\ldots N_{h}\}$ denotes the number of hidden units in the layer *h*^*th*^ of the original model, and ℑ denotes the total number of hidden units in the original model, i.e., $ℑ=\{N_{1}+N_{2}+N_{3}+\ldots N_{h}\}$.
-
$C_{h}=\{C_{1},C_{2},C_{3},\ldots C_{h}$ } denotes the number of hidden units in the layer *h*^*th*^ in the optimized model based on optimization and $\Delta =\sum_{i=1}^{h}C_{i}$.
-
Q denotes the original deep neural network model with *h* number of hidden layers, and the set of weights is represented by *Ψ*.
- ℜ denotes the optimized deep neural network model, and the set of its weights is denoted by W with the constraint that W⫅Ψ.
- *A*_*N*_ denotes the accuracy of *N*_*th*_ client, and it is computed using the following equation (refer to Eq 2).
  $$\begin{array}{c} A_{N}=\frac{\text{No.}\;\text{of}\;\text{correctly}\;\text{predicted}\;\text{sample}\;\text{by}\;N^{th}\text{client}}{\text{Total}\;\text{number}\;\text{of}\;\text{samples}\;\text{pass}\;\text{to}\;N^{th}\;\text{client}} \end{array}$$ (2)

In this article, we aim to find the ℜ which must be a minimal subset of *Ψ* with the constraint that the performance of ℜ should be near Q over the test dataset. Three major objectives have been considered, which ensure that the performance of the optimized model is near that of the original model. Our first objective is the maximization of average accuracy in an FL environment with *N* clients, represented using Eq 3. This objective ensures that the optimized model has the highest average accuracy on *N* clients.

$$\begin{array}{c} \text{Objective}\;\text{1}:\text{Max}\frac{\sum_{i=1}^{N}A_{i}}{N} \end{array}$$ (3)

The second objective is to minimize the average loss in the network. Therefore, we have added the losses of individual clients and divided the sum by the number of clients. We have thus taken into account the input of every client to determine the optimal structure of the base model. The second objective is presented in Eq 4.

$$\begin{array}{c} \text{Objective}\;\text{2}=\text{Min}\frac{\sum_{i=1}^{N}\pi_{i}}{N} \end{array}$$ (4)

The third objective is the minimization of the number of hidden units in the base model. Minimizing hidden units helps us to improve the inference time as well as reduce storage space. It helps us to improve the inference time as fewer operations have taken place due to the fewer hidden units. If fewer operations are there in a deep neural network, then computational time is also less. Moreover, due to the smaller number of hidden units, less storage is required to store the trained model (refer to Eq 5).

$$\begin{array}{c} \text{Objective}\;\text{3}=\text{Min}\;\frac{\Delta }{ℑ} \end{array}$$ (5)

There are two objectives that we have to minimize and a third objective that we have to maximize. In an optimization problem either we have to maximize or minimize so to put all objectives in the same scale we have converted the first objective for minimization by reducing from 1, i.e., Min (1- $\frac{\sum_{i=1}^{N}A_{i}}{N}$). After using the weighted sum approach, we have combined all three objectives and derived a final objective, which is represented in Eq 6.

$$\begin{array}{c} \text{Objective}=\text{Min}\;\{w_{1}\times \left(1-\frac{\sum_{i=1}^{N}A_{i}}{N}\right)+w_{2}\times \left(\frac{\sum_{i=1}^{N}\pi_{i}}{N}\right)+W_{3}\times \left(\frac{\Delta }{ℑ}\right)\} \end{array}$$ (6)

where, *w*_1_ + *w*_2_ + *w*_3_ = 1. The above objective is utilized as a fitness function in a genetic algorithm to find the optimal base model, i.e., ℜ.

## 4 Proposed methodology

In this section, a discussion about federated learning (FL), the use of federated learning for sports classification, and the generation of an optimal base model for FL are discussed. In the next subsection, a brief discussion about federated learning is presented.

### 4.1 Introduction to federated learning

**Fig 1:**
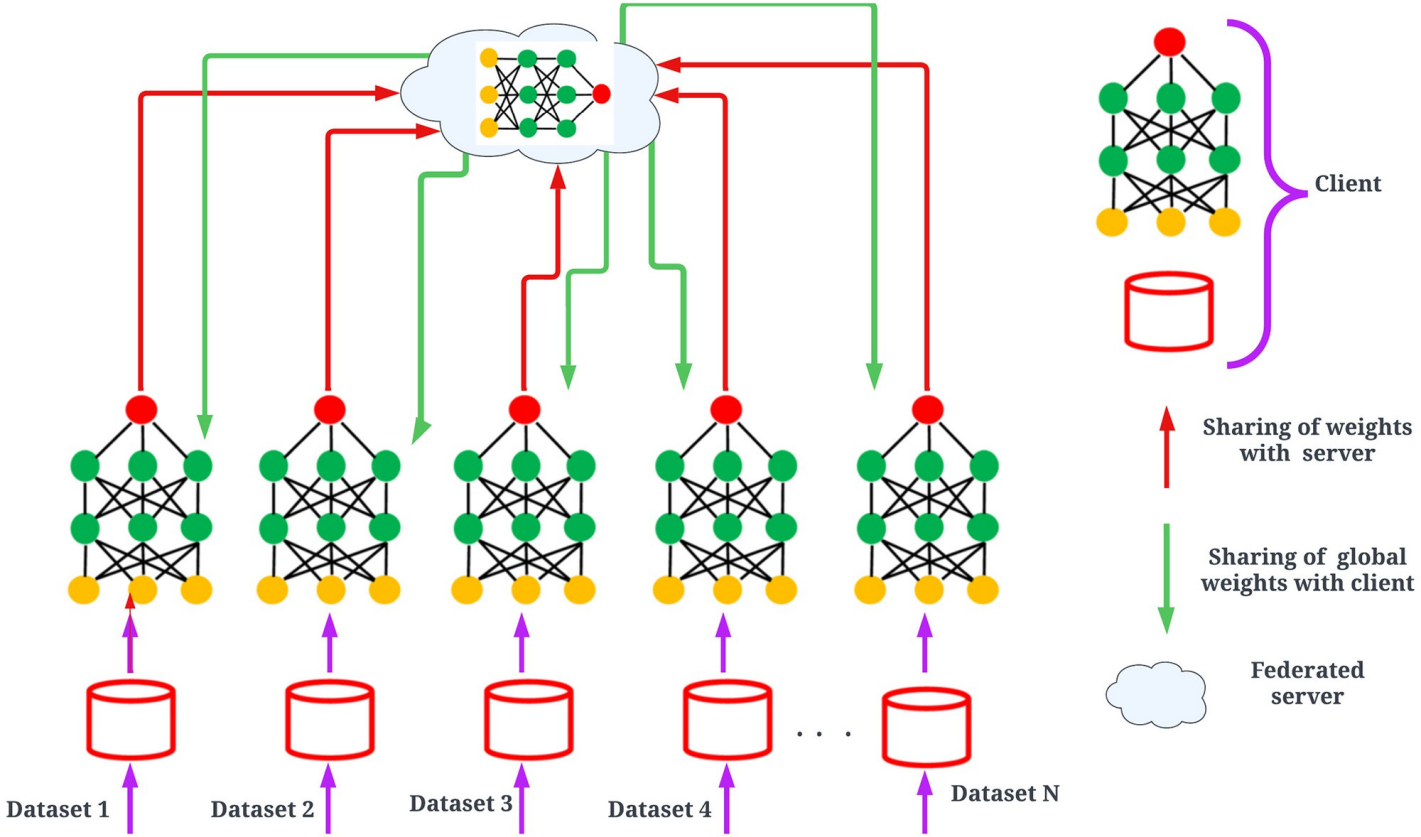
A general architecture of federated learning model.

Federated learning is a special type of artificial intelligence technique that enables the training of machine and deep learning algorithms in decentralized data sources, such as IoT devices, without transferring the data to a central server [35–37]. The pictorial representation of federated learning is presented in Fig 1.

From the Fig 1, it is clearly visible that there are *N* diverse datasets which passed through the *N* different computing devices. These devices process the respective data and share the computed weights with the central server. Moreover, on each computing device, the same deep learning architecture has been deployed. The role of the central server is to generate the aggregated model and share the aggregated model back with the devices. This process is continued until the performance of the aggregated model is not up to the mark or as per the user’s requirements [38, 39].

### 4.2 Federated learning model generation using global averaging

In federated learning, the global model updates the weight using the federated average method. In this method, the weight of the global model is updated using the average value of the client’s weight [36]. A graphical representation is shown in Fig 1 where the red color line shows the weight sharing to the server and the green line represents the updated weight sharing to the client for the second round of communication.

The aggregation typically involves taking the average of the model parameters from the different devices or servers. This averaging process helps to combine the knowledge learned from the various data sources while preserving privacy. Without having direct access to the raw data, the central server may make use of the local models’ combined intelligence by averaging them [37].

In federated learning, global averaging ensures that the final global model combines the knowledge acquired from many devices or servers, making it more reliable and representative [40]. Additionally, it helps to reduce the effects of potential biases in the specific local models. The formula for global averaging may be shown as follows in mathematics (refer to Eq 7) [40, 41].

$$\begin{array}{c} \theta_{global}=\left(w_{1}\times \theta_{1}+w_{2}\times \theta_{2}\ldots +w_{n}\times \theta_{n}\right)/\left(w_{1}+w_{2}+...+w_{n}\right) \end{array}$$ (7)

where:

- *θ*_*global*_ denotes the global model’s parameter.
- *θ*_1_, *θ*_2_, …, *θ*_*n*_ denote the parameters of the models from each client.
- *w*_1_, *w*_2_, …, *w*_*n*_ denotes weight assign to individual client.

The weights *w*_1_, *w*_2_, …, *w*_*n*_ are commonly decided depending on elements like the volume of data on each device or the computing power of each device. The weights may, for instance, be inversely proportional to the processing resources or proportional to the amount of data samples. By using the weighted average, the contributions from each device or server are included in the overall model, enabling a collaborative and privacy-preserving learning process [42]. It’s important to note that the specific formula for global averaging may vary depending on the federated learning framework or algorithm being used. Different approaches may use different weighting schemes or aggregation methods [37, 40].

### 4.3 Generation of an optimal base model for FL

Here, a discussion about the use of a genetic algorithm (GA) for the generation of an optimal base model is presented. In the first section, we have discussed the genetic algorithm and its intermediate operations, and in the next sub-section, the discussion of the use of the genetic algorithm for optimizing the base model is presented with suitable examples.

#### 4.3.1 Introduction to GA

**Fig 2:**
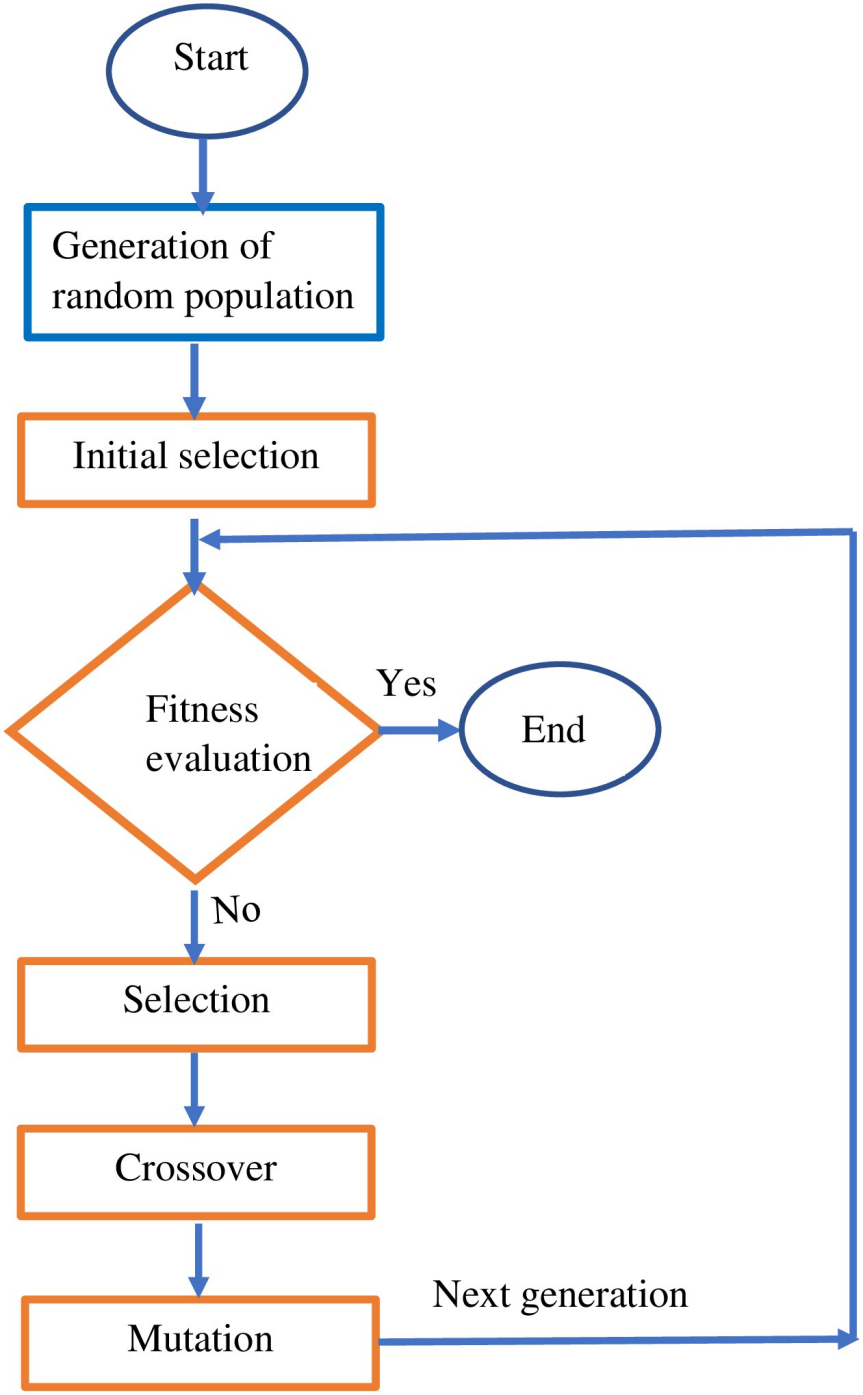
Flowchart of genetic algorithm.

Genetic Algorithm (GA) is one of the oldest optimization and search techniques, inspired by natural selection [43, 44]. Moreover, it is also known as a search technique as it searches for the optimal solution from the provided search space by performing the intermediate operations [45]. The flowchart of the genetic algorithm is presented in Fig 2.

The process of GA starts with the generation of the initial population, which is also known as the collection of chromosomes. Generally, chromosomes are generated randomly, and they are the valid solution to a given problem. In the proposed work, the length of the chromosomes is constant, which is equal to the number of hidden units in a deep neural network, and after the intermediate operations of GA, there is no change in the length of the chromosomes [46, 47].

In GA, selection, crossover, and mutation are the major three intermediate activities. After the generation of chromosomes, a selection operation takes place to identify strong chromosomes based on the fitness value. A higher fitness value indicates that chromosomes are strong, and strong chromosomes always generate stronger chromosomes after the execution of crossover and mutation operations [48]. All the intermediate operations, i.e., selection, crossover, and mutation, are executed until the termination criteria is not met [45].

#### 4.3.2 Use of GA for the development of an optimized base model

Here, a discussion about using GA to find the optimal structure of the base model in a federated learning environment is presented. In the previous section, we discussed that the GA process started with the generation of the initial population, which is also known as the pool of chromosomes. Therefore, first, we discuss the generation of chromosomes.

**Fig 3:**
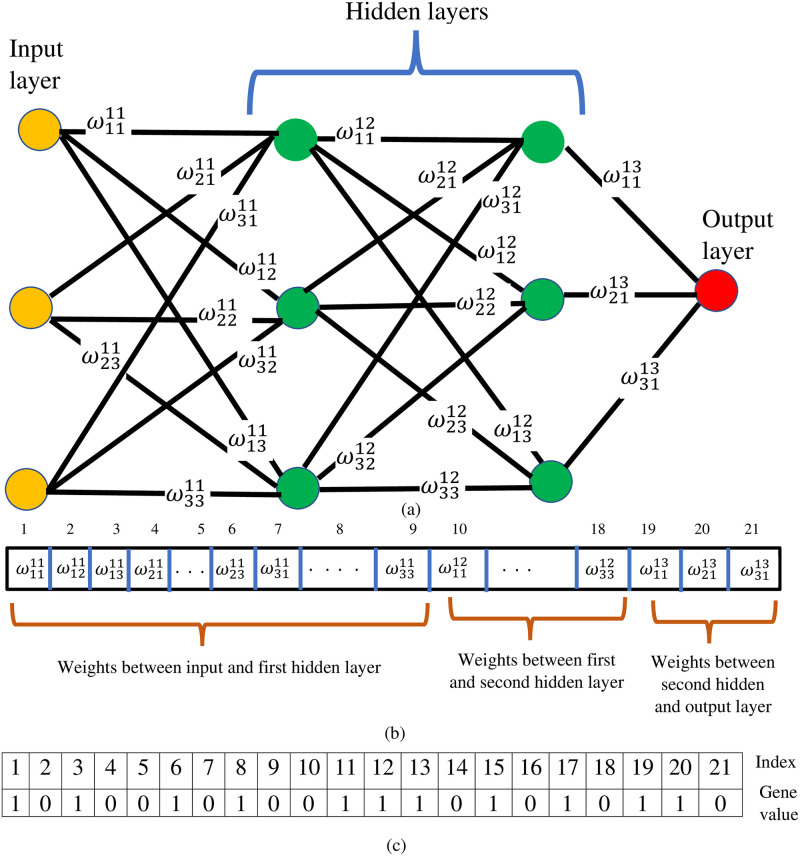
(a) Sample architecture of the deep neural network, which consists of 4 layers including input and output (b) Representation of weights of the deep neural network presented in a part using vector (c) Chromosome for deep neural network presented in part an in binary.

*Chromosome representation*. In the proposed work, chromosomes are generated randomly, and the length of the chromosomes is equal to the number of hidden units in the deep neural network or base model. Moreover, there is no change in the length of the chromosomes after performing the other intermediate operations. In part (a) of Fig 3, a neural network is presented that consists of two hidden layers with three hidden units in each layer. From the figure, it is visible that there are 21 weights in the network i.e., $\omega_{11}^{11}\ldots \omega_{13}^{31}$, and all the weights are presented in the form of a vector (refer to part (b)).

In presentations that have been vectorized, we first insert all of the weights between the input and the first hidden layer, and then we place the weights between the first hidden and second hidden layers in the vector. The process continues until all the weights are processed and placed in the array. After presenting all the weights in a vectorized way, we generated the chromosomes randomly by placing the random binary values in a vector with a length equal to the number of hidden units. A sample chromosome is presented in Fig 3 part (c). The corresponding neural network architecture for this chromosome is presented in Fig 3 part (a). In any chromosome, value 0 represents that the corresponding weight is not considered, and vice versa in the final architecture of the model.

In the proposed work, for the implementation of a genetic algorithm for minimizing the architecture of the base model, 500 chromosomes have been generated and 20% chromosomes are selected. Moreover, for the computation of fitness value, we have used the formula presented in Eq 6 with the Roulette Wheel selection algorithm [49] to select the strong chromosomes.

**Fig 4:**
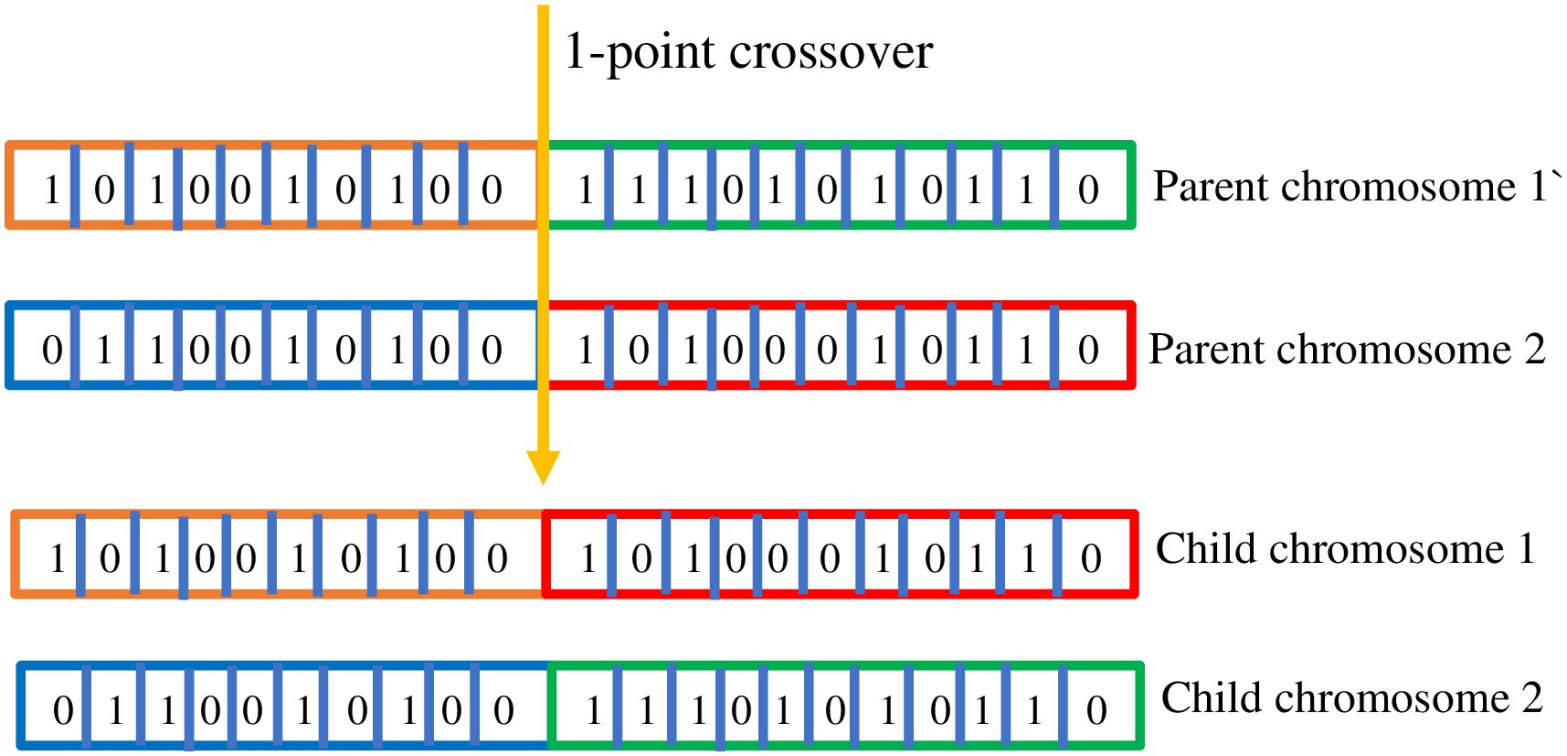
Crossover operation.

*Crossover*. After the generation of a pool of chromosomes, crossover operations have been performed. Crossover is also known as reproduction or biological crossover. In a crossover operation, two parents’ chromosomes exchanged information and created two child chromosomes. There are various methods to apply the crossover, but we have applied the 1-point crossover operation. In Fig 4, an example of a crossover operation has been presented. Moreover, after performing the crossover operation, there are 4 chromosomes (2 child & 2 parent), and based on fitness value, two chromosomes out of four are discarded and the rest two join the pool of population. Crossover operation helps to find the optimal solution quickly, as after every crossover operation, GA only adds the better chromosomes to the population pool [50, 51].

**Fig 5:**
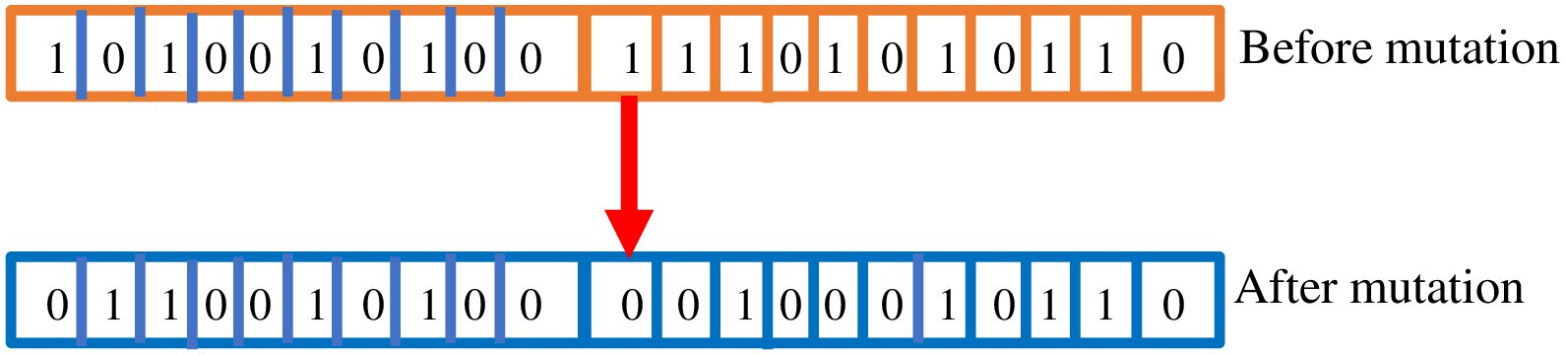
Mutation operation.

*Mutation*. After performing the crossover operation, the mutation is another important intermediate operation in the genetic algorithm. In simple terms, we can define the mutation as a small tweaking in the chromosome for getting a new chromosome [52]. The mutation process helps GA to achieve quick convergence of the algorithm, and it is applied with low probability. Moreover, mutation is also related to the exploration of the search space. There are various methods, i.e., bit flip, random resetting, inversion, etc., to apply the mutation. In our proposed work, we have applied the bit-flipping method to mutation. In the bit-flipping approach, we have randomly selected a gene, and its value is flipped. Here, flipping means that if the gene value is 0 then the changed value is 1, and vice versa. In the proposed work, our goal is to reduce the size of the base model; hence, we tried to turn the 1s into 0s during mutation. The mutation process is depicted visually in Fig 5.

*Termination criteria*. For the generation of an optimal base model, selection, crossover, and mutation operations are executed until termination criteria is satisfied to achieve the higher fitness score. Moreover, we have used the termination criteria in such a way that the difference between the fitness scores of the top two chromosomes is less than 0.0001. The values of hyper parameters used in GA are presented in Table 3.

**Table 3:** Parameter used in genetic algorithm.

Parameter used in the study	Value or range
Randomly generated chromosome	500
Selection algorithm	Roulette wheel
Crossover	1-point
Mutation	0.1%–0.7%
Termination criteria	Difference between score of best two chromosomes ≤ 0.0001

### 4.4 Performance evaluation metrics

Especially in deep learning and information retrieval, binary classification tasks frequently employ the F1 score, recall, and accuracy measurements. By taking into account many facets of a model’s predictions, they aid in evaluating its performance. Ratio of true positive predictions (TP) and the total number of true positive predictions and false positive predictions (FP) made by the model is called the precision of the model. A mathematical expression is shown in Eq 8.

$$\begin{array}{c} precision=TP/(TP+FP) \end{array}$$ (8)

On the other hand, recall is the ratio of true positives to the total number of true positive predictions and false negative (FN) predictions. In Eq 9, a mathematical expression of recall is shown.

$$\begin{array}{c} recall=TP/(TP+FN) \end{array}$$ (9)

F1-score is another important parameter to test the model performance, and it is the harmonic mean of precision and recall. A mathematical expression is given in Eq 10.

$$\begin{array}{c} F1=[2*((Precision*Recall)/(Precision+Recall))] \end{array}$$ (10)

AUC-ROC is one of the important matrices that validates the performance of deep learning models. The high area under the curve denotes better performance, while the lower area indicates a less reliable model. The ROC plot includes a true positive rate (TPR) and a false positive rate (FPR). In Eqs 11 and 12 show the mathematical representation of FPR, TPR respectively.

$$\begin{array}{c} FPR=FP/(FP+TN);\;\text{where}\;\text{FP}=\;\text{False}\;\text{positive}\;\text{and}\;\text{TN}=\;\text{True}\;\text{Negative.} \end{array}$$ (11)

$$\begin{array}{c} TPR=TP/(TP+FN);\text{where}\;\text{TP}=\;\text{True}\;\text{positive}\;\text{and}\;\text{FN}=\;\text{False}\;\text{Negative.} \end{array}$$ (12)

## 5 Experimental results

All the experiments were performed on the NVIDIA DGX V-100 system, which features eight NVIDIA Tesla P100 GPUs, each with 16GB of memory, for a total of 128GB of GPU memory. The system also includes two Intel Xeon E5–2698 v4 CPUs, 512GB of RAM, and 7.68TB of SSD storage. For the code development, the Python programming language has been used. Different libraries of Python, such as Keras, Tensorflow, and Matplotlib, have been extensively explored for the computation of the results.

In the proposed federated learning model, we have executed four famous deep learning architectures, namely AlexNet, VGG19, ResNet50, and EfficientNetB3, as the base model in FL. The major reason for the use of these pre-trained architectures is data scarcity in the proposed work. For experimental work, a sports image dataset has been used (refer to Table 2 in section 1 to know more about the dataset) and samples are equally shared with all the FL clients for training, validation, and testing purposes. AlexNet is one of the popular deep convolutional neural networks promoted by Geoffrey Hinton and Alex Krizhevsky in 2012 [53]. Moreover, the architecture won the title of the famous image recognition challenge named ILSVRC in 2012 by achieving state-of-the-art performance on the ImageNet dataset [54].

**Fig 6:**
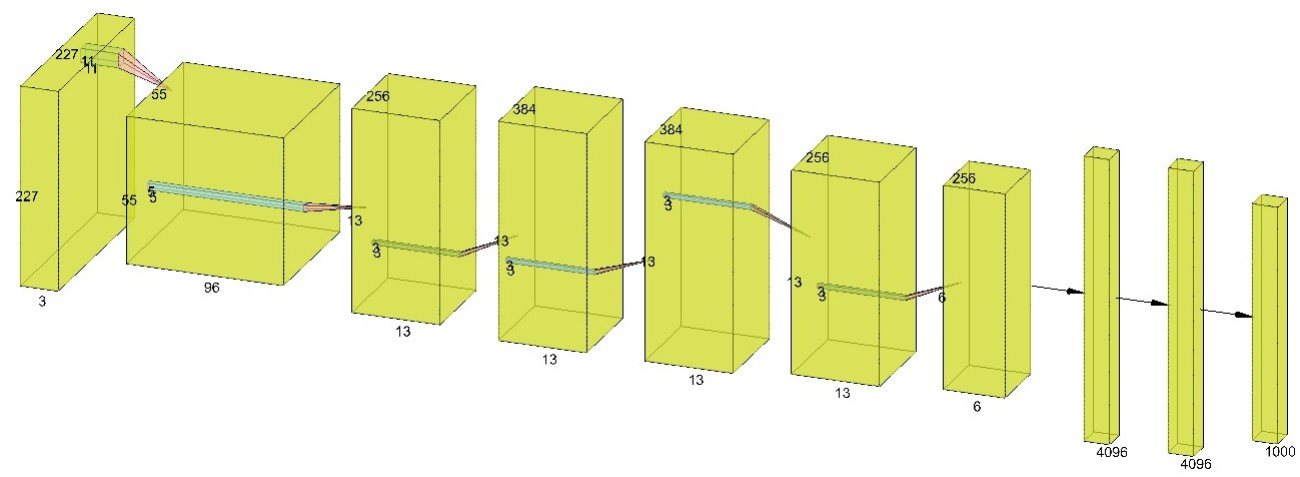
Architecture of AlexNet.

AlexNet comprises eight layers, out of which five are for convolution operations and three are fully connected, with over 50+ million parameters. The architecture of AlexNet is provided in Fig 6.

**Fig 7:**
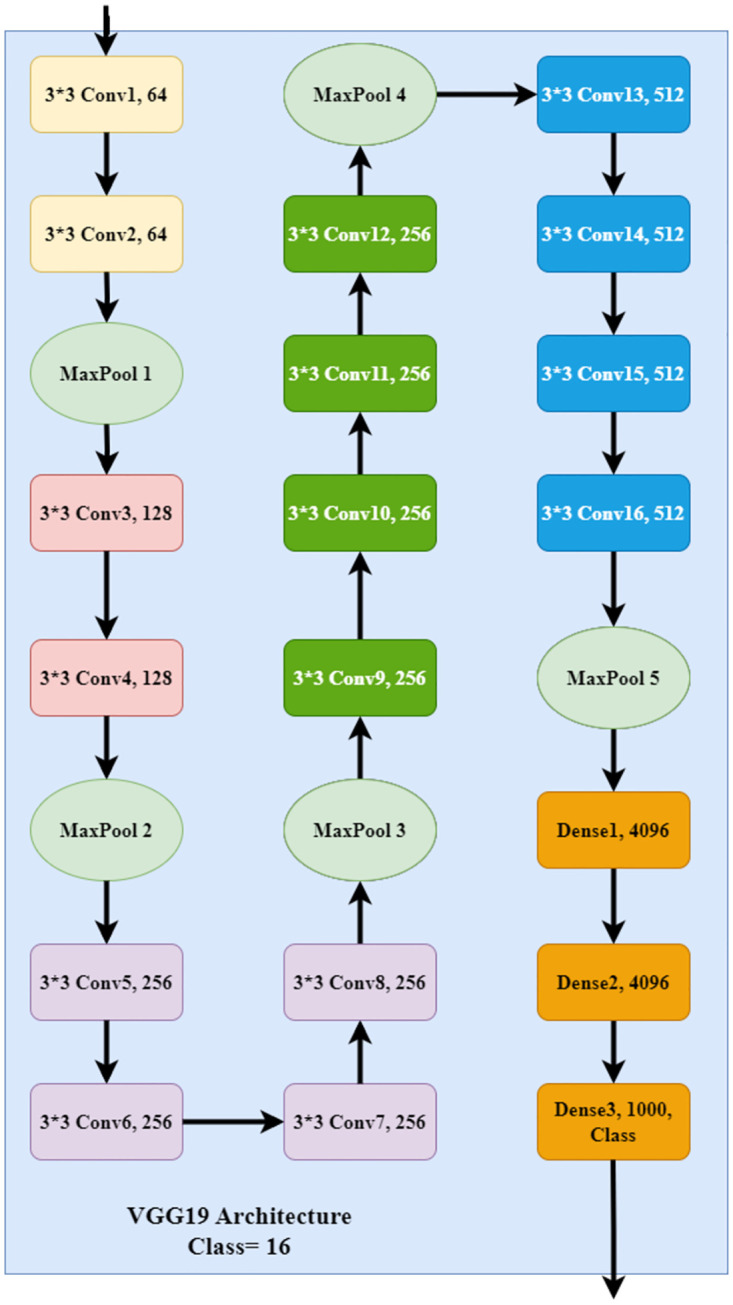
Architecture of VGG19.

VGG19 (Visual Geometry Group) is another popular deep learning model architecture that has 19 layers and is very popular after AlexNet. It has 16 convolution layers along with five max pooling and three fully connected dense layers with 4096 nodes [55]. The pictorial representation of the VGG19 architecture is presented in Fig 7, where all the layers are represented with different colors. The input image shape in VGG19 is 224*224*3 for the RGB image, and it uses a (3*3) kernel along with a 1 pixel stride size. In VGG19, spatial padding is used to preserve the spatial resolution of images. All the max-pooling is performed over a 2*2 pixel window with stride 2 [56].

**Fig 8:**
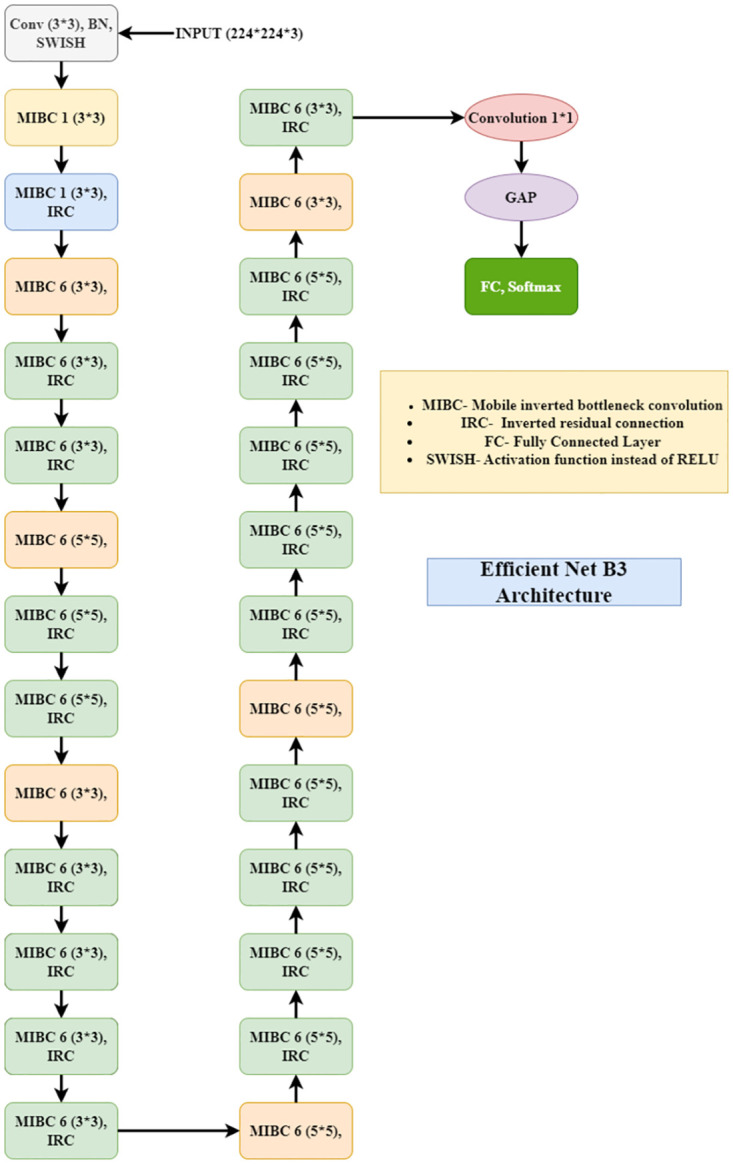
Architecture of efficientNetB3.

EfficientNet is another architecture that is known as a better version of the ResNet18 model [57]. A model can be scaled up either depth-wise or width-wise. It was also random, and a deep neural network was sometimes required to take the input of a larger image as input and make it have better accuracy. EfficientNet can take large images as input, and it uses a special technique called compound coefficient to scale up the model to reach higher accuracy. This compound technique helps to scale the model uniformly from all sides instead of randomly width- or depth-wise. It uses AutoML and the scaling method to achieve better accuracy by scaling up uniformly. This architecture uses an inverted bottleneck convolution, which is similar to MobileNetV2, but it is much larger due to the increase in FLOPS, which helps scale up the base model of EfficientNet [58]. The schematic diagram of an efficient net is shown in Fig 8.

ResNet is also a deep learning architecture that can have a variable size depending on how big each of the layers is. In this architecture, each layer has a 3*3 convolution layer followed by a max pooling layer. It consists of stem blocks and finally fully connected layers [59].

**Fig 9:**
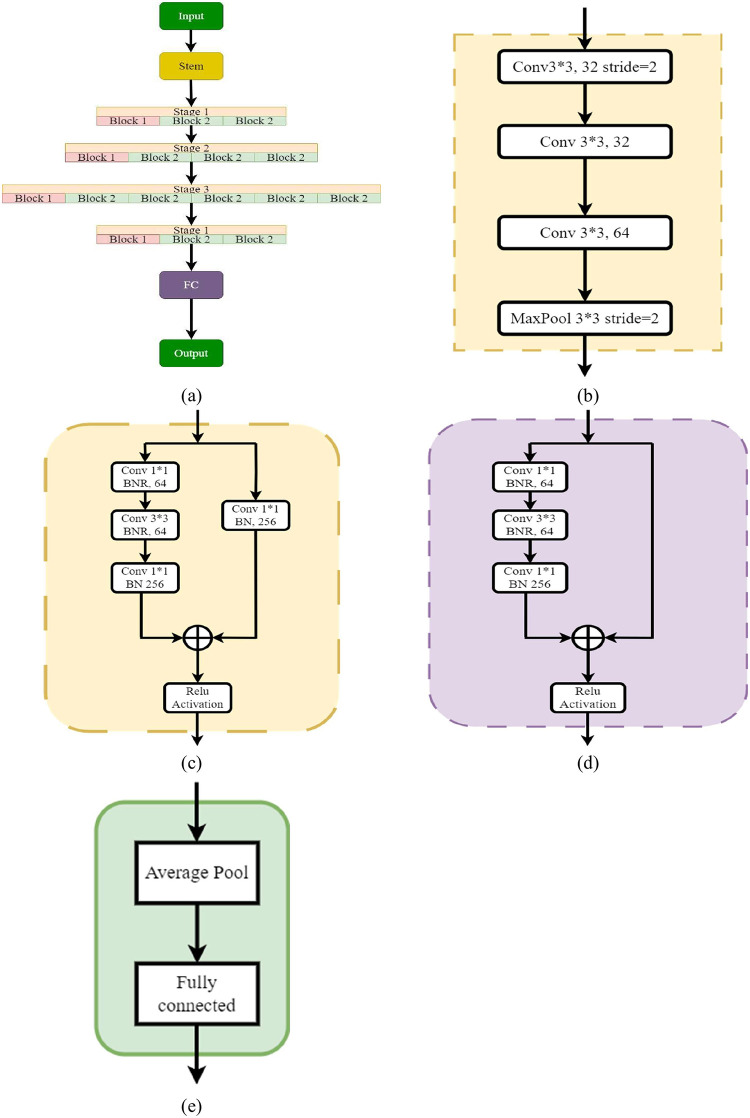
Architecture of ResNet50. a) ResNet50 architecture; b) Stem block; c) Block1-Stage 1; d) Block2-Stage 1; e) FC Block.

The schematic diagram of ResNet50 is shown in Fig 9.

In this article, we consider four models, and each model runs with several clients (i.e., 2, 4, 6, 9, and 10). A complete list of hyperparameters related to FL is shown in Table 4 and unbalanced & balanced datasets have been passed in each model for experimental work.

**Table 4:** Parameter used in federated learning model.

Parameters in FL	Range or values
Client	{2, 4, 6, 9,10}
Communication round	250
Iteration per communication round	5
Epoch	100
Learning rate	0.001
Optimizer	Adam
Batch size	32

In federated learning, the server distributes the model, random weight pair to the clients. Upon receiving the model, random weight pair, each client locally trains the model using their private dataset. As a result of the training, different new weights are produced by individual clients which are later shared by the clients to the server. As and when the server receives weights from each client, it computes the average of the received weights for fitness evaluation (we assume that the server initiates the process of weight averaging only when it receives weights from each client). The average weight value is again shared by the server to the client. Since in FL the server holds a validation dataset, the fitness of the model depends on the average weight computed by the server in each communicated round using the Eq 6. However, the communication round stops as and when the fitness measured in the current round is found to be greater than the previous round.

**Fig 10:**
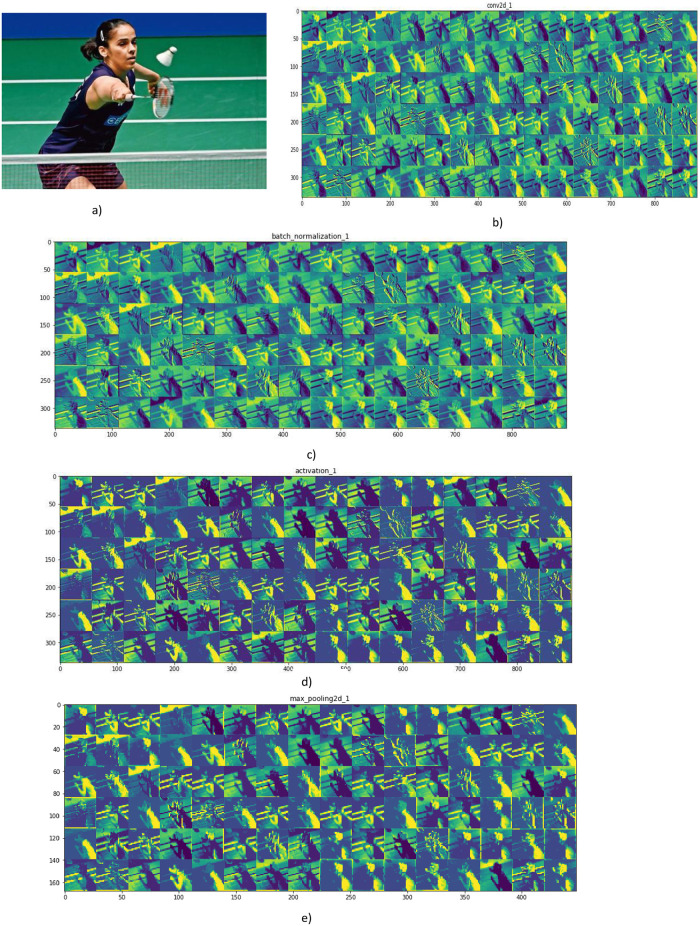
(a) Input image; (b) Activation values after the first convolution operation; (c) Activation values after the batch normalization operation; (d) Activation values at convolution layer 2; (e) Activation values after the max-pooling operation.

Activation values indicate the data that is kept in the hidden layers. As we know, a convolutional neural network is the combination of a convolutional layer, a max-pooling layer, and a fully connected layer. The activation values for the AlexNet architecture over the balanced sports dataset is presented in Fig 10.

**Fig 11:**
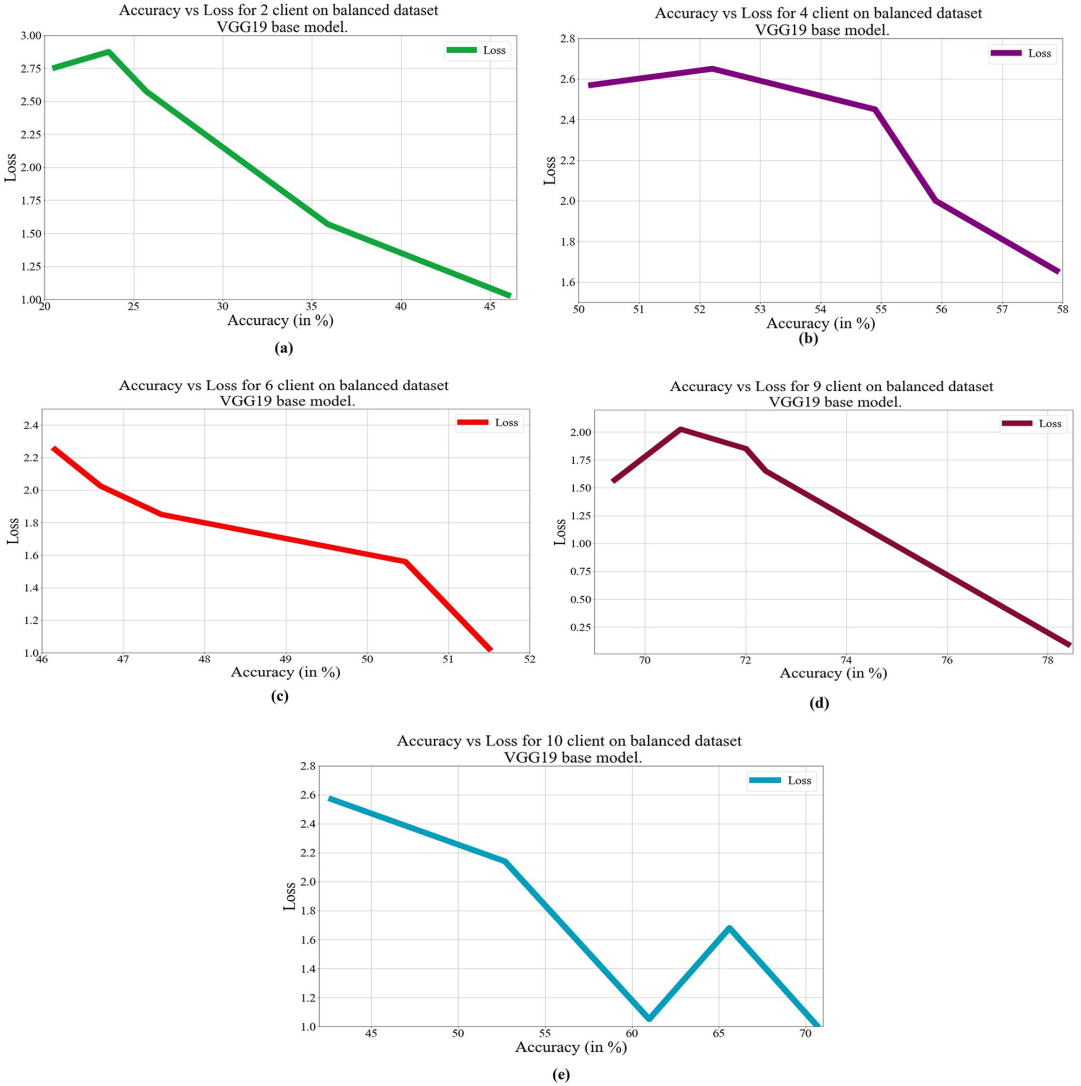
Loss vs. Accuracy with the VGG19 base model over a balanced data set: a) 2 clients, b) 4 clients, c) 6 clients, d) 9 clients, e) 10 clients.

**Fig 12:**
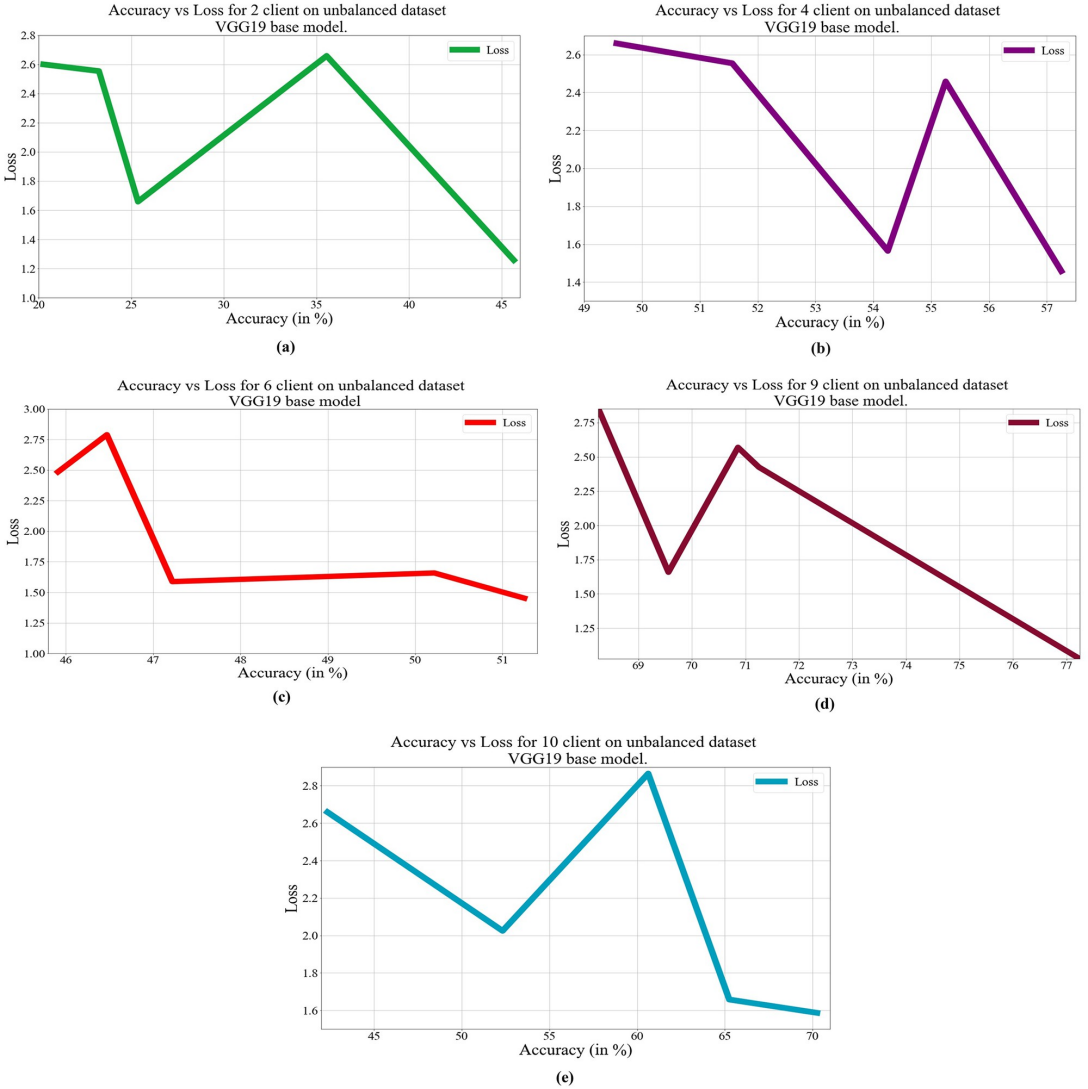
Loss vs. Accuracy with the VGG19 base model over an unbalanced data set: a) 2 clients, b) 4 clients, c) 6 clients, d) 9 clients, e) 10 clients.

**Fig 13:**
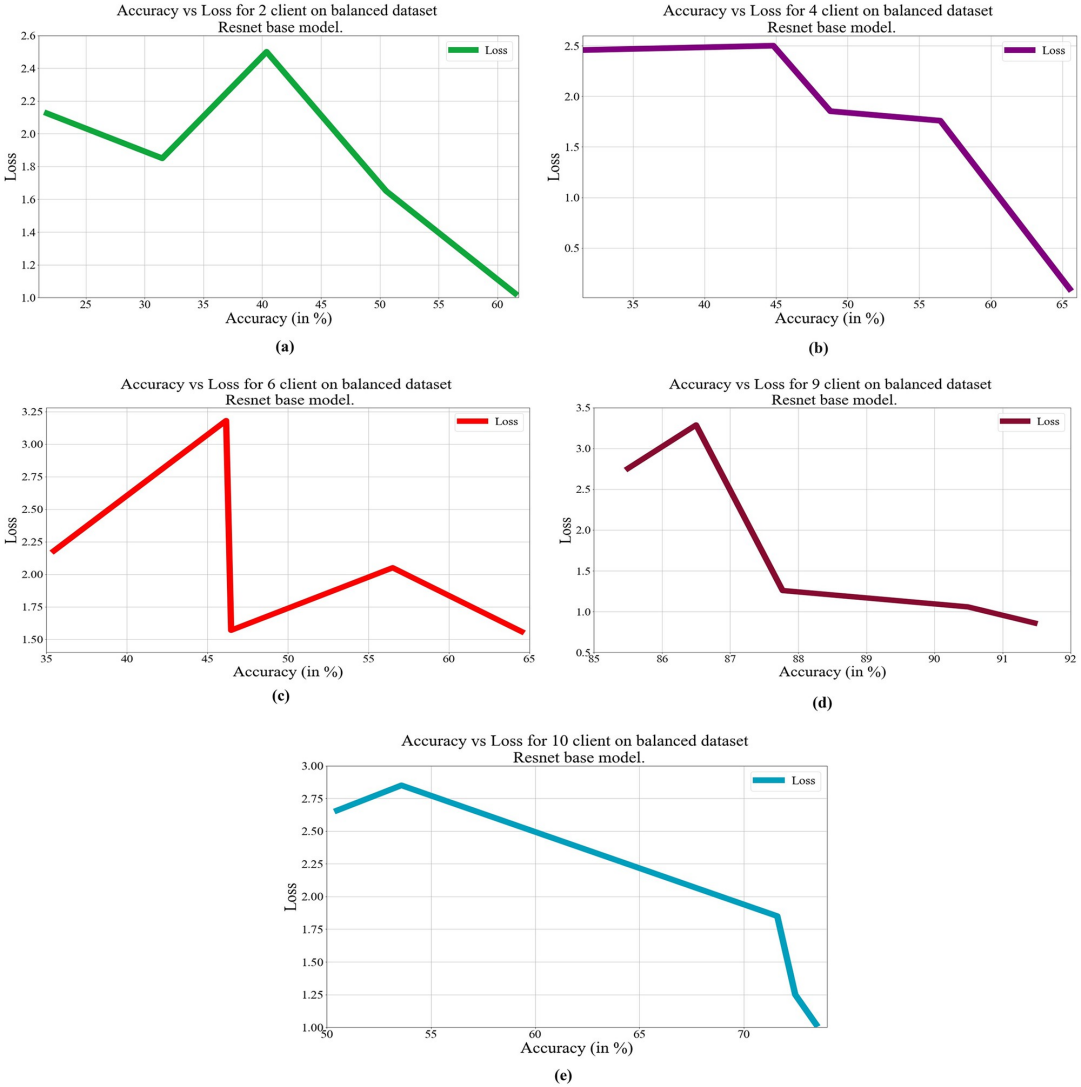
Loss vs. Accuracy with the ResNet50 base model over a balanced data set: a) 2 clients, b) 4 clients, c) 6 clients, d) 9 clients, e) 10 clients.

**Fig 14:**
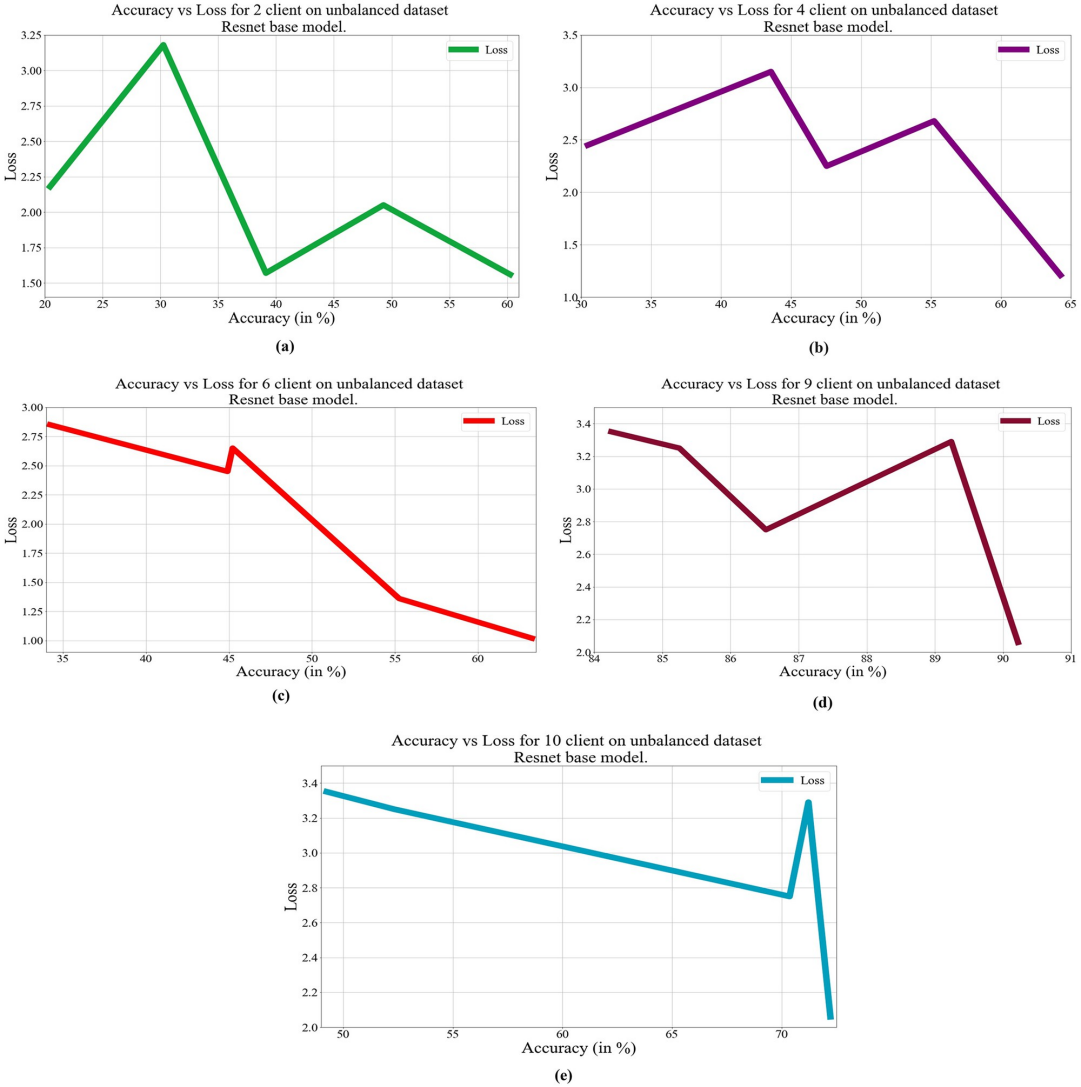
Loss vs. Accuracy with the ResNet50 base model over an unbalanced data set: a) 2 clients, b) 4 clients, c) 6 clients, d) 9 clients, e) 10 clients.

**Fig 15:**
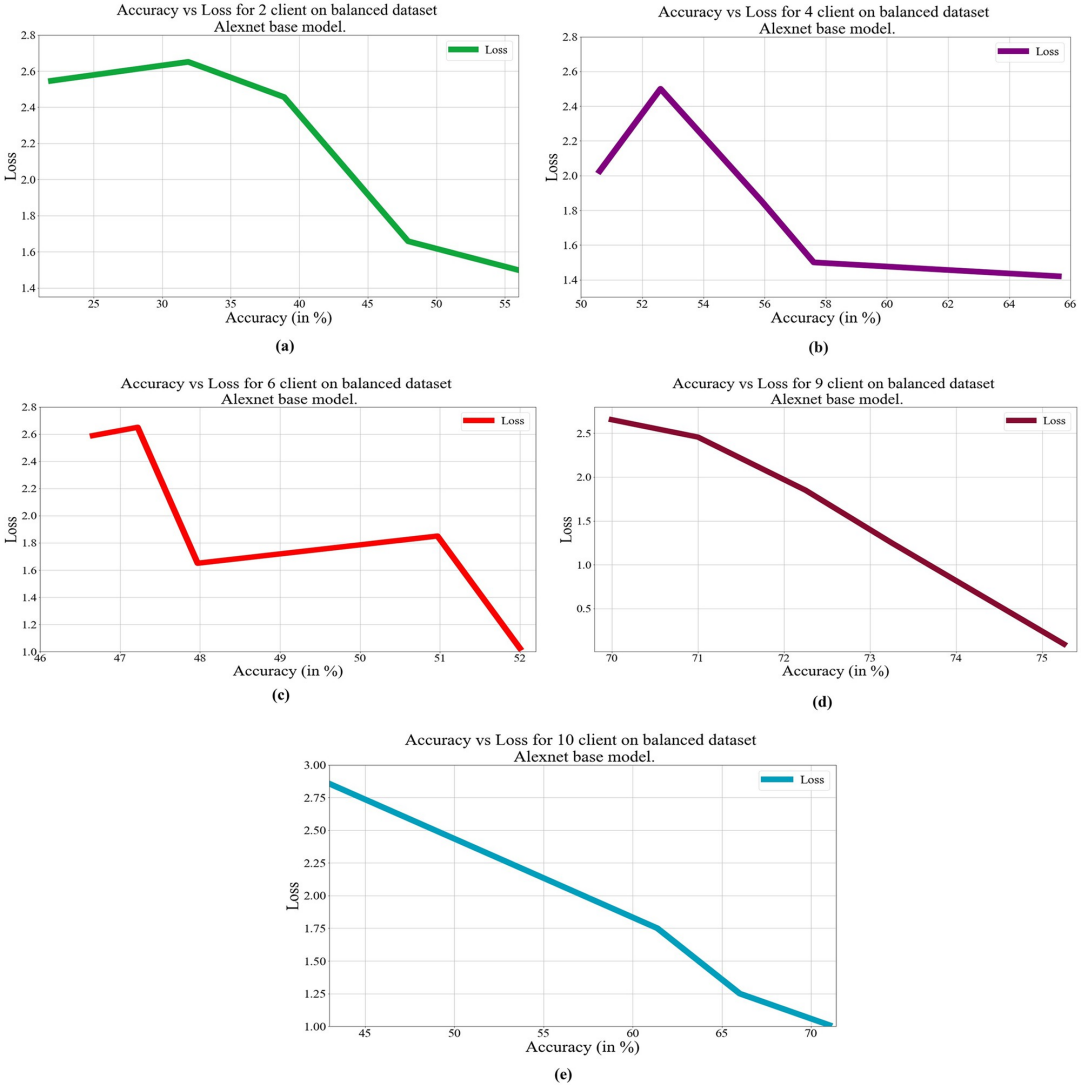
Loss vs. Accuracy with the AlexNet base model over a balanced data set a) 2 clients; b) 4 clients; c) 6 clients; d) 9 clients; e) 10 clients.

**Fig 16:**
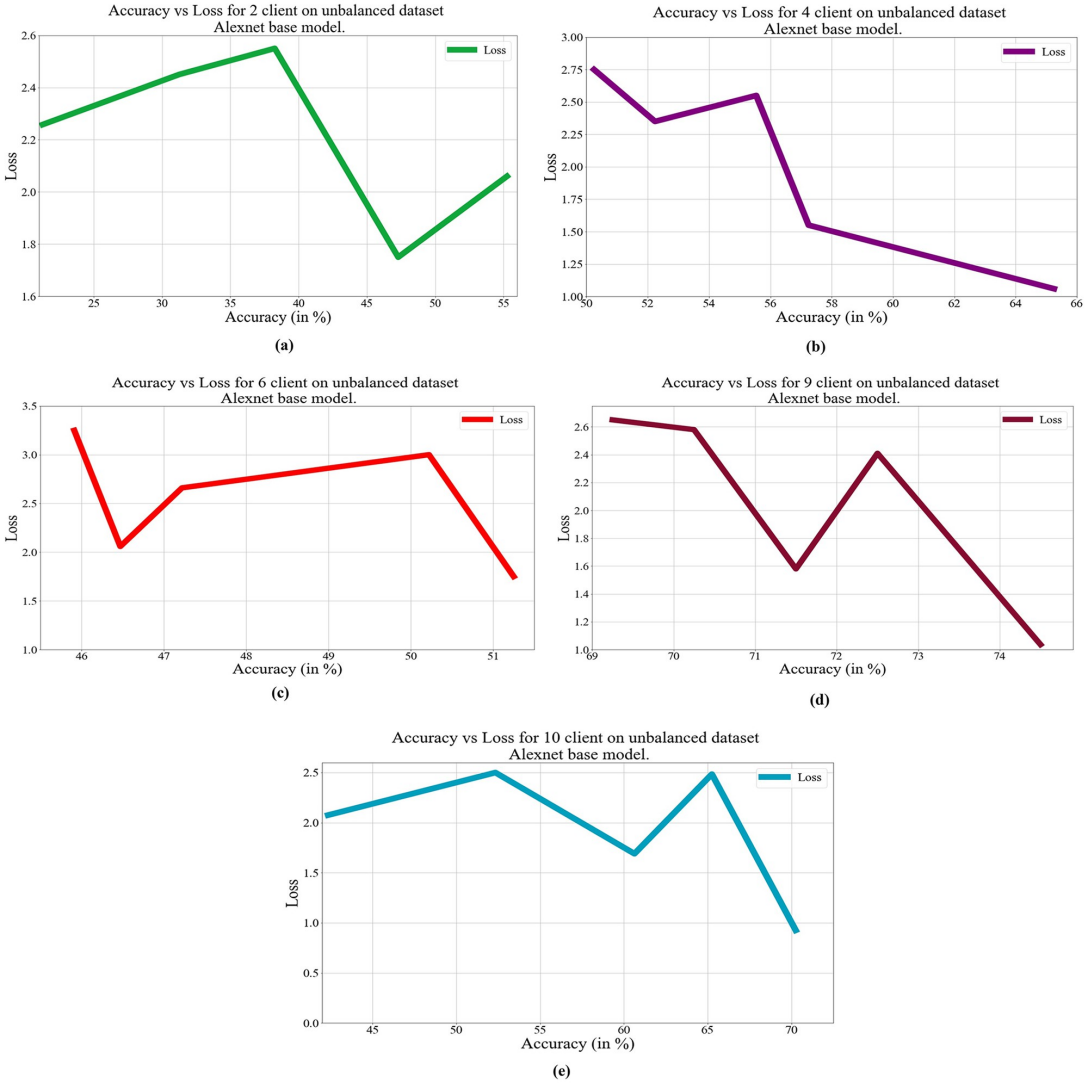
Loss vs. Accuracy with the AlexNet base model over an unbalanced data set: a) 2 clients, b) 4 clients, c) 6 clients, d) 9 clients, e) 10 clients.

**Fig 17:**
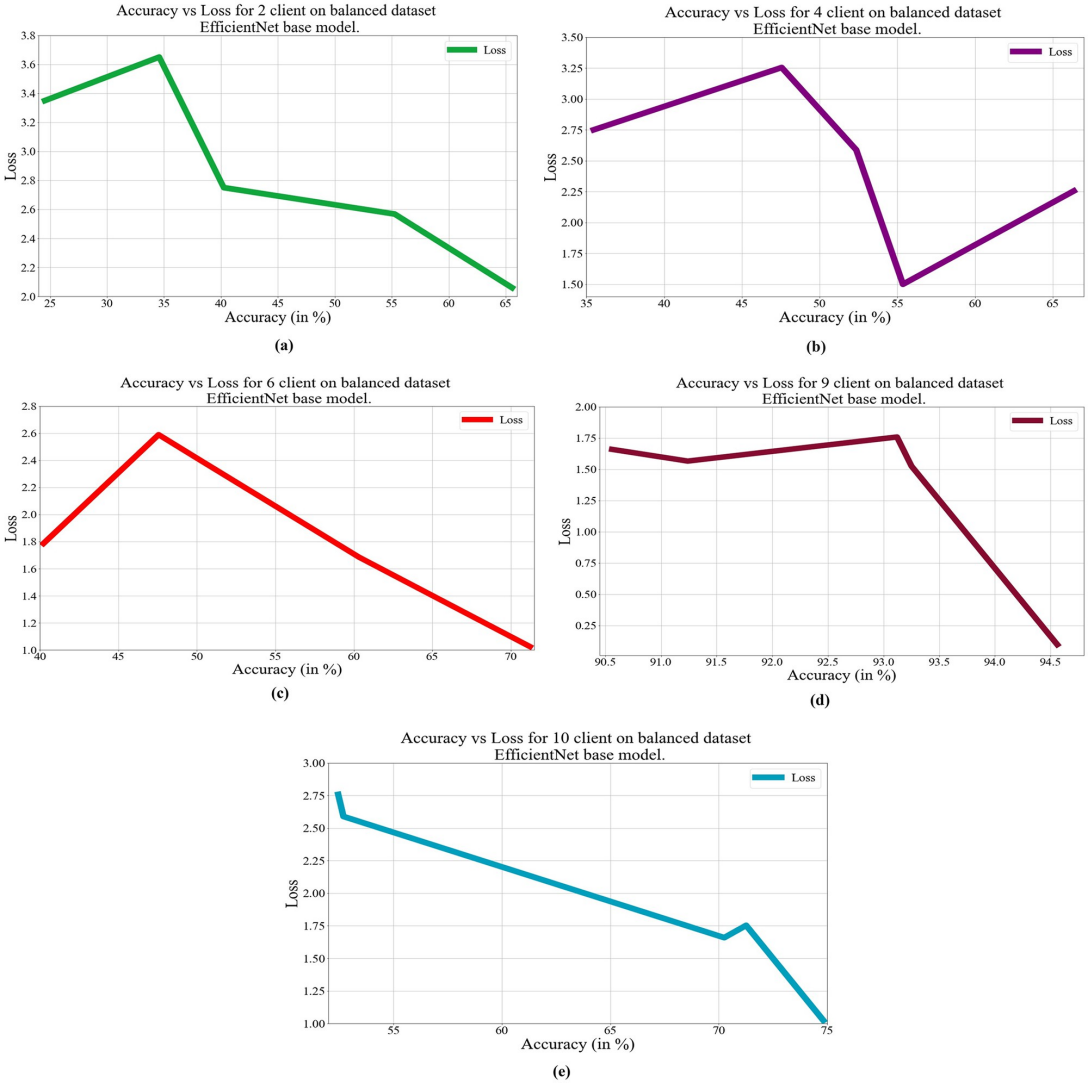
Loss vs. Accuracy with the EfficientNetB3 base model over a balanced data set a) 2 clients, b) 4 clients, c) 6 clients, d) 9 clients, e) 10 clients, and an unbalanced dataset e) 2 clients, f) 4 clients, g) 6 clients, h) 9 clients, i) 10 clients.

**Fig 18:**
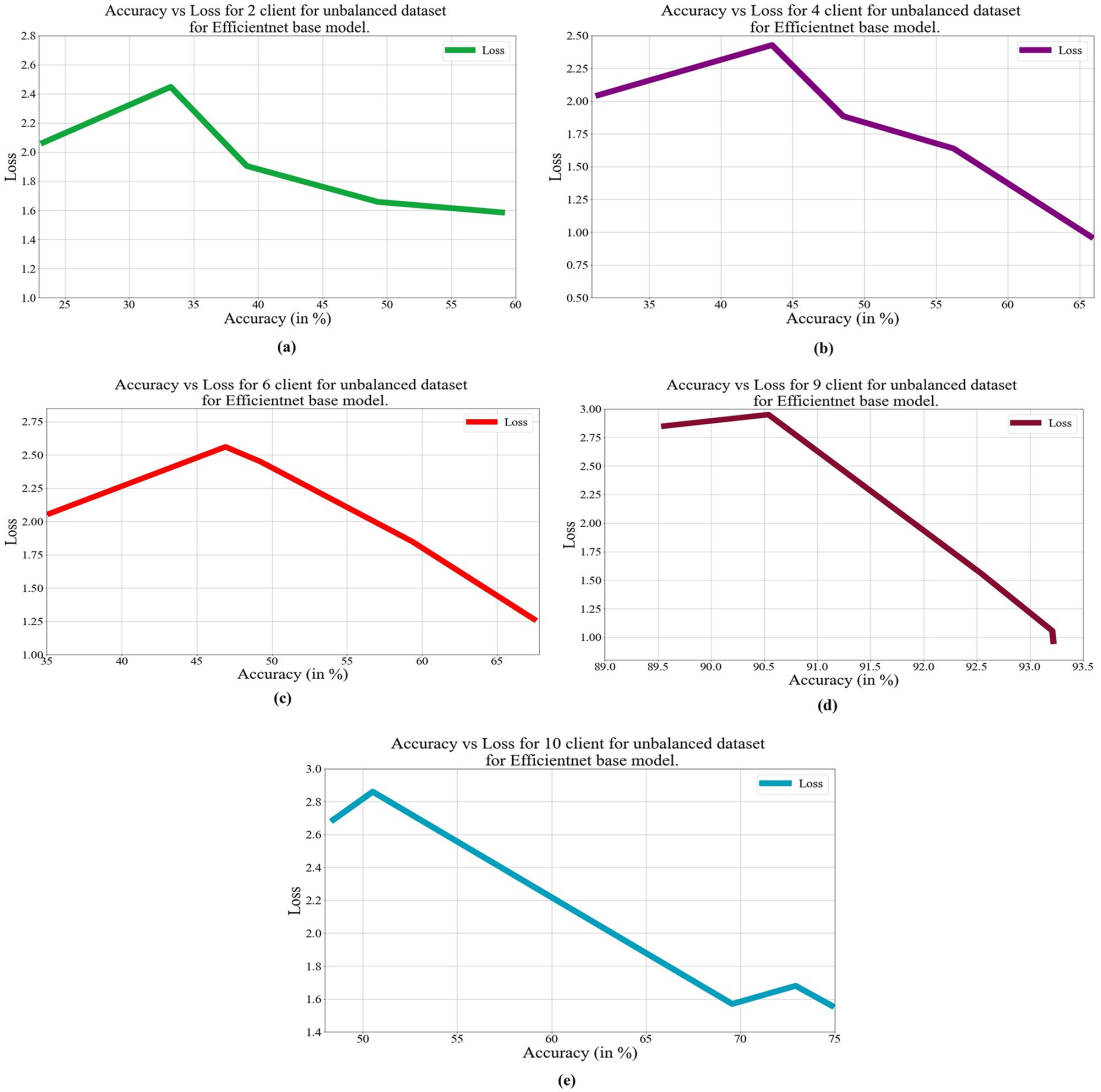
Loss vs. Accuracy with the EfficientNetB3 base model over an unbalanced data set a) 2 clients; b) 4 clients; c) 6 clients; d) 9 clients; e) 10 clients.

In deep learning, loss, and accuracy are both crucial measures. By calculating the difference between forecasts and actual values, loss aids in learning, whereas accuracy offers a general indicator of correctness. Academicians and practitioners may iteratively enhance the performance of their models by tracking and optimizing these measures. The loss vs. accuracy for different architectures as base models over balanced and unbalanced datasets is presented in Figs 11–18. Data values for Figs 11 and 14 are provided in the S1 Table (see S1 Table). Moreover, we have also computed the training time in both cases: 1) global model generation using the averaging method and 2) development of an optimized global model using GA. The results are provided in Table 5.

**Table 5:** Time spent to train the various base architecture models in federated learning using the proposed methodology and global averaging.

# Clients	Model	Training time (in seconds)	
		FL with global averaging	Proposed approach
2	AlexNet	8848	11520
4		15598	26852
6		16998	35246
9		17563	39656
10		18559	45696
2	VGG19	9854	12659
4		10558	13256
6		11557	15696
9		15555	16989
10		15545	189789
2	ResNet50	7854	13569
4		8857	15696
6		10255	14787
9		11245	12569
10		11285	15236
2	EfficientNetB3	6854	7589
4		7968	8958
6		7854	10225
9		8696	11457
10		9698	147588

**Fig 19:**
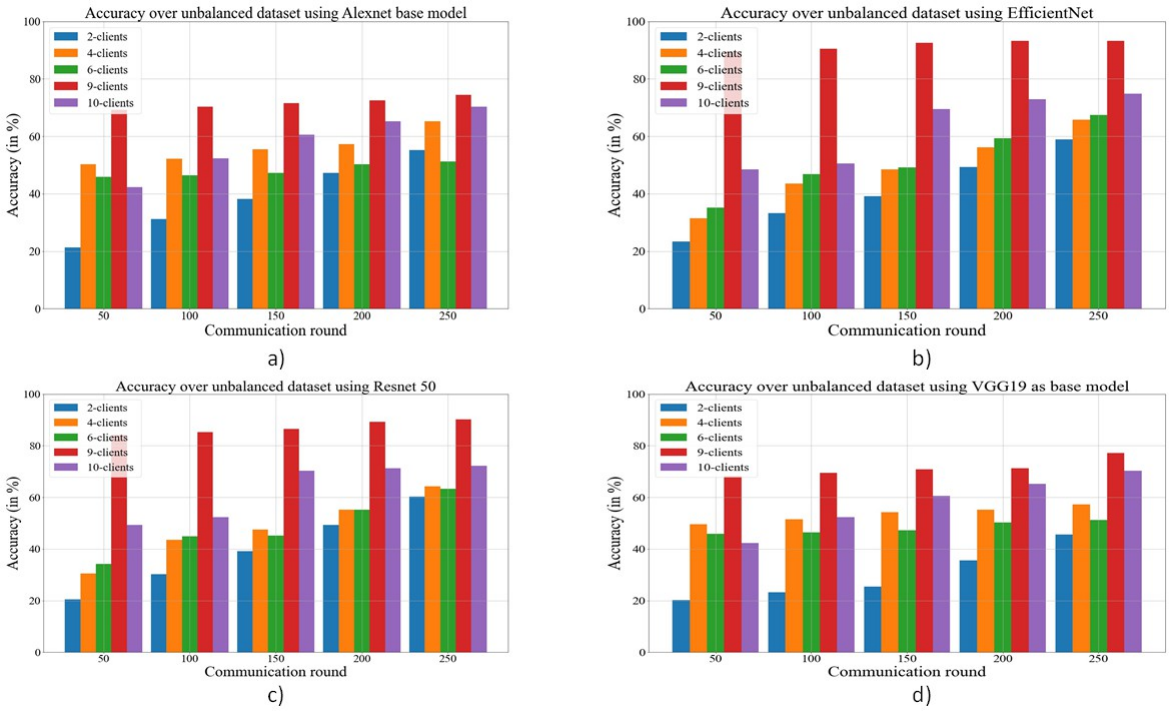
Comparison of accuracy for an unbalanced dataset using different deep learning models as the base model in federated learning. a) AlexNet as the base model; b) EfficientNetB3 as the base model; c) ResNet50 as the base model; d) VGG19 as the base model.

An indicator of how well a deep learning model predicts class labels is accuracy, a commonly used and straightforward statistic. It is useful for model selection and comparison, tracking model performance during training, and determining how effectively the model generalizes to new data. As accuracy is an important metric to check the performance of deep learning models we calculate the accuracy over balanced and unbalanced data sets. In Fig 19, the accuracy is shown for different base models and also for different numbers of clients. In the case of an unbalanced data set, EfficientNetB3 gives the best accuracy for 9 clients. Table 6 has a summary of F1-Score, recall, and precision for various models that is deployed against balanced and unbalanced data sets.

**Table 6:** A tabular representation of Recall, Precision, F1-Score for unbalanced and balanced dataset for all used model.

Model	Unbalanced Dataset			Balanced Dataset		
	F1-Score	Recall	Precision	F1-Score	Recall	Precision
AlexNet	0.84	0.89	0.89	0.84	0.90	0.88
VGG19	0.89	0.88	0.87	0.89	0.88	0.87
ResNet50	0.90	0.89	0.88	0.91	0.90	0.90
EfficientNetB3	0.91	0.91	0.91	0.92	0.91	0.93

**Fig 20:**
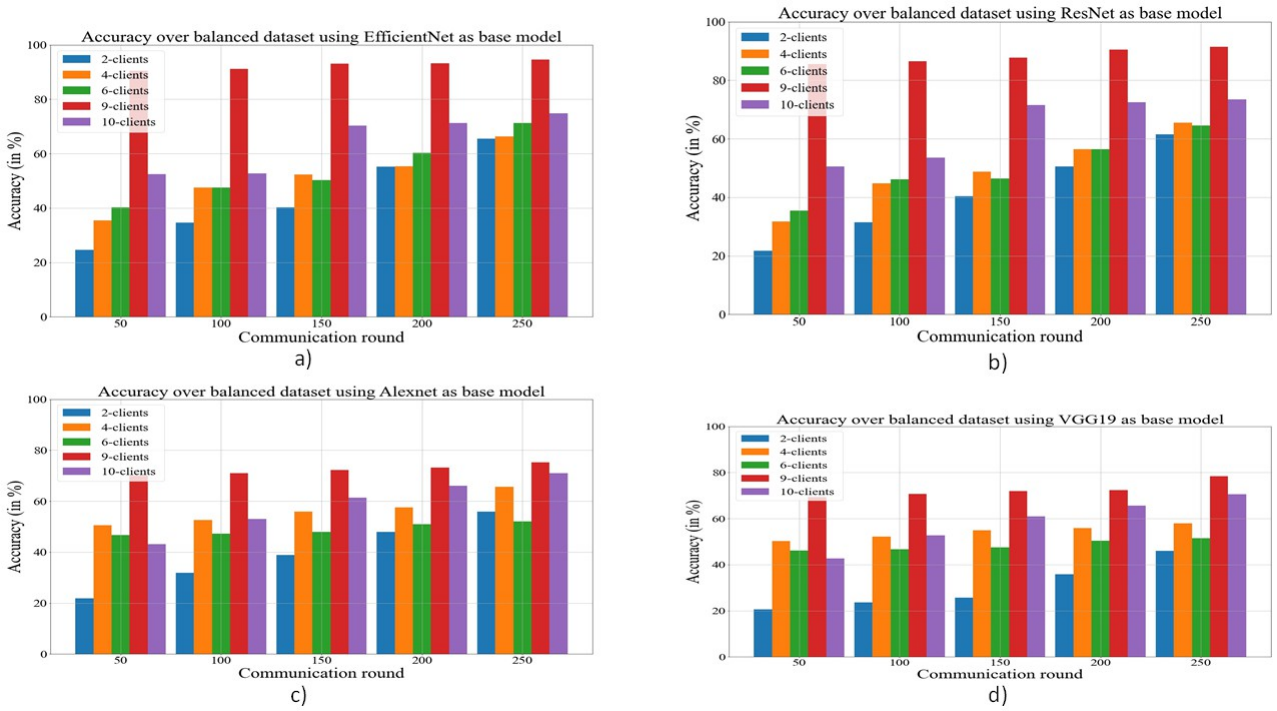
Comparison of accuracy for a balanced dataset using different deep learning models as the base model and federated learning. a) EfficientNetB3 as the base model; b) ResNet50 as the base model; c) AlexNet as the base model; d) VGG19 as the base model.

During experiments, the data set is randomly assigned among the clients in both cases. In the proposed work, the number of clients has varied from 2 to 10, and both types of data sets (balanced and unbalanced) have been used for performance evaluation purposes. The accuracy with respect to communication round and number of clients over an unbalanced and balanced dataset is presented in Figs 19 & 20 respectively.

From Figs 19 & 20, it has been visible that maximum accuracy has been achieved by the proposed methodology over a balanced dataset with 10 clients.

**Fig 21:**
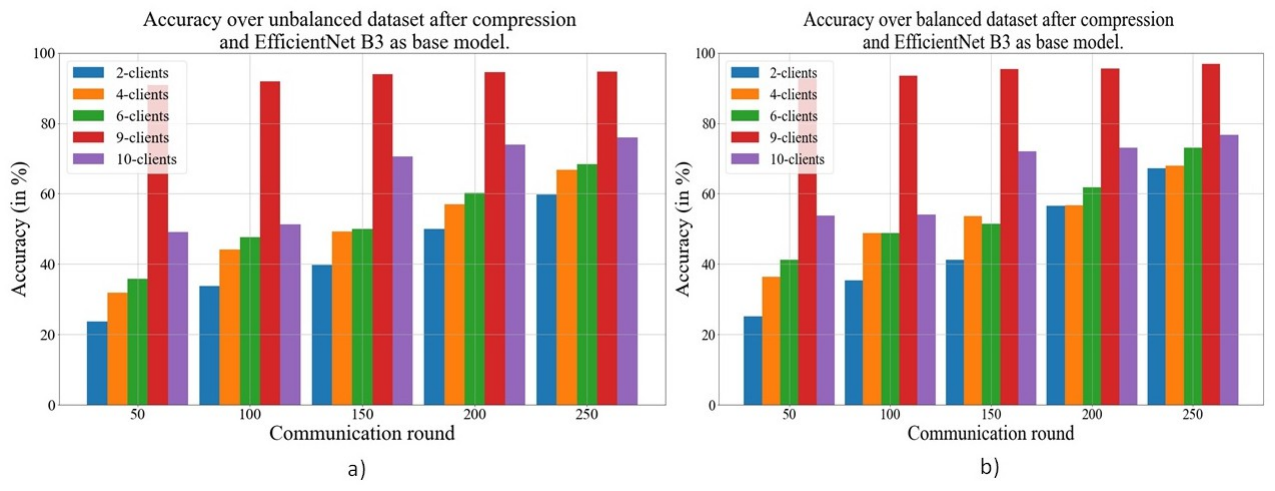
Comparison of accuracy after compression using GA and using EfficientNetB3 as the base model a) Unbalanced b) Balanced data set.

In Fig 21, the accuracy comparison between FL with global averaging and the proposed algorithm on the balanced and unbalanced data sets is presented, and it is visible that the proposed algorithm performs better as compared to the global averaging approach. The main reason behind the same is that the proposed algorithm always selects a set of existing weights for which the accuracy is highest. Moreover, intermediate operations such as crossover and mutations also help the algorithm achieve better performance quickly.

**Fig 22:**
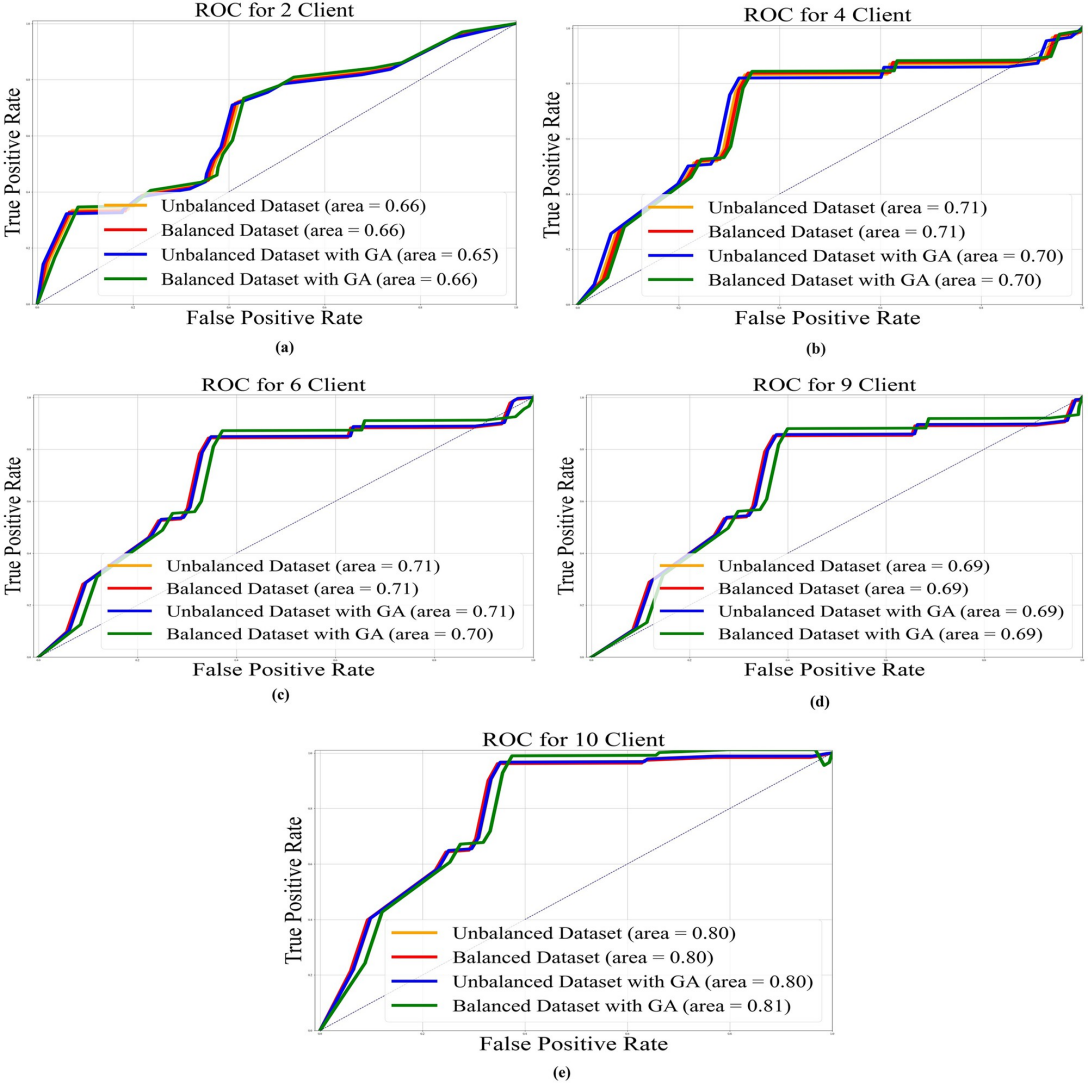
AUC-ROC with area under curve for VGG19 base model: a) 2 clients; b) 4 clients; c) 6 clients; d) 9 clients; e) 10 clients.

In Figs 22–Fig 25, the ROC curve is shown with the AUC value for AlexNet, EfficientNetB3, ResNet50, and VGG19, respectively, and it also shows the AUC for balanced and unbalanced datasets with and without GA for all models.

**Fig 23:**
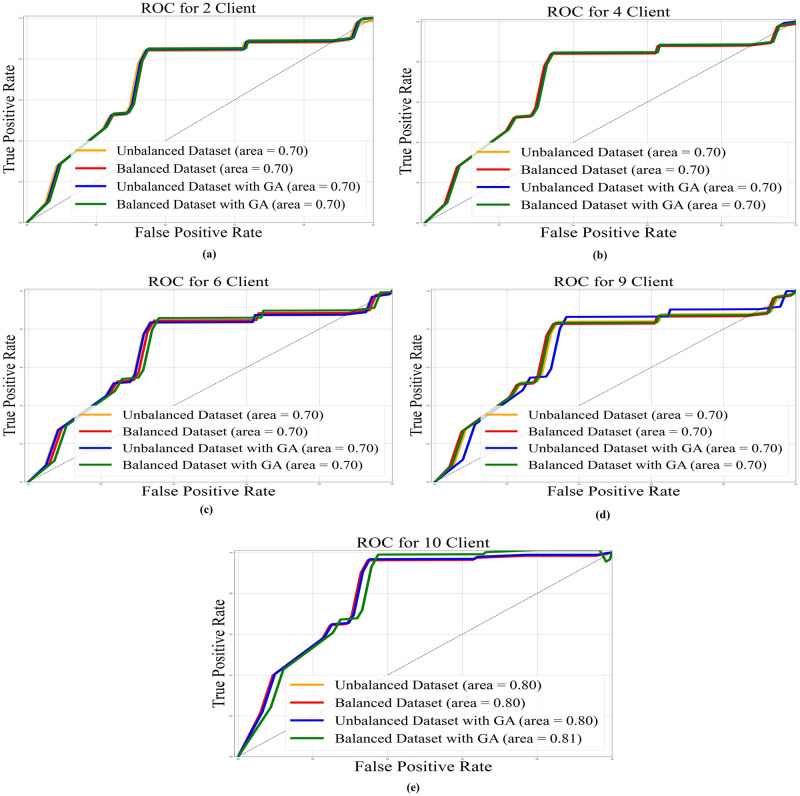
AUC-ROC with an area under curve for ResNet50 base model: a) 2 clients; b) 4 clients; c) 6 clients; d) 9 clients; e) 10 clients.

**Fig 24:**
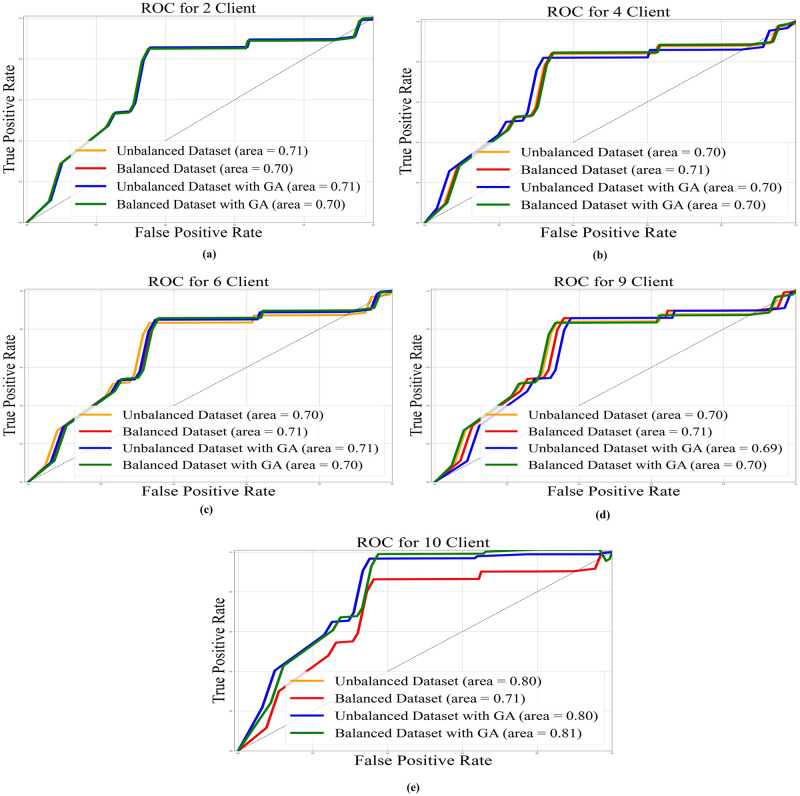
AUC-ROC with area under curve for AlexNet base model: a) 2 clients; b) 4 clients; c) 6 clients; d) 9 clients; e) 10 clients.

**Fig 25:**
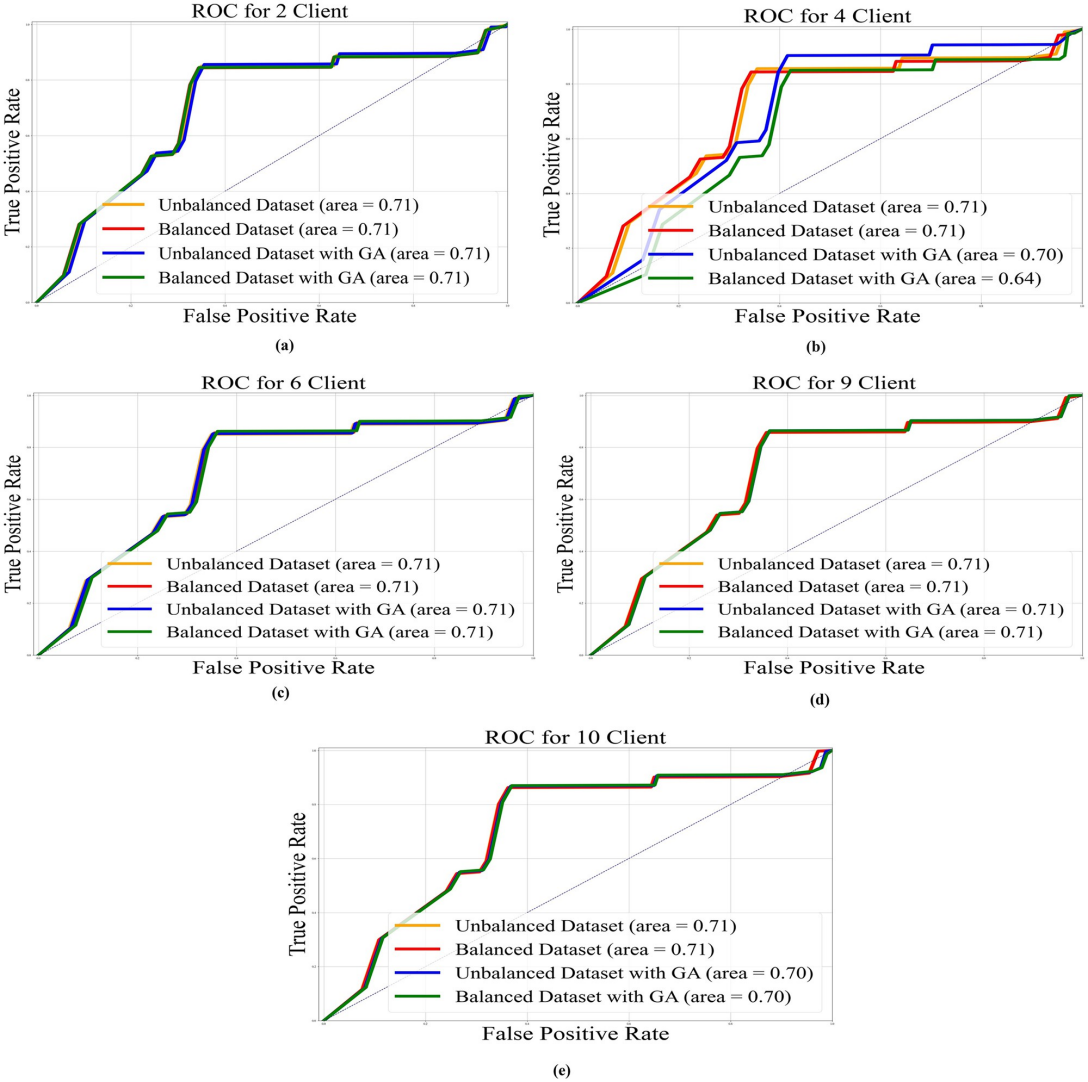
AUC-ROC with area under curve for EfficientNetB3 base model: a) 2 clients; b) 4 clients; c) 6 clients; d) 9 clients; e) 10 clients.

We have also applied the proposed approach to different datasets (Potato [60], tomato [61] and Indian food [62]) to check the efficacy of the proposed approach and results under the different performance evaluation metrics are presented in Table 7.

**Table 7:** Accuracy, F1 Score, precision, recall for other datasets with proposed algorithm over balance and unbalanced dataset.

Model	Dataset	Unbalanced				Balanced			
		F1-score	Recall	Precision	Accuracy	F1-score	Recall	Precision	Accuracy
AlexNet	Tomato	.84	.89	.90	.88	.82	.89	.87	.88
	Potato	.81	.81	.84	.78	.87	.74	.78	.79
	Food	.62	.68	.77	.75	.64	.68	.68	.67
ResNet50	Tomato	.85	.86	.86	.87	.84	.89	.91	.94
	Potato	.84	.84	.84	.82	.86	.85	.86	.84
	Food	.71	.78	.78	.79	.75	.79	.78	.77
VGG19	Tomato	.87	.90	.90	.845	.85	.88	.86	.85
	Potato	.81	.87	.77	.75	.84	.86	.86	.89
	Food	.75	.74	.75	.74	.71	.88	.87	.77
EfficientNetB3	Tomato	.77	.87	.72	.75	.92	.88	.88	.87
	Potato	.79	.81	.81	.89	.91	.89	.77	.76
	Food	.75	.74	.77	.76	.89	.89	.87	.77

**Fig 26:**
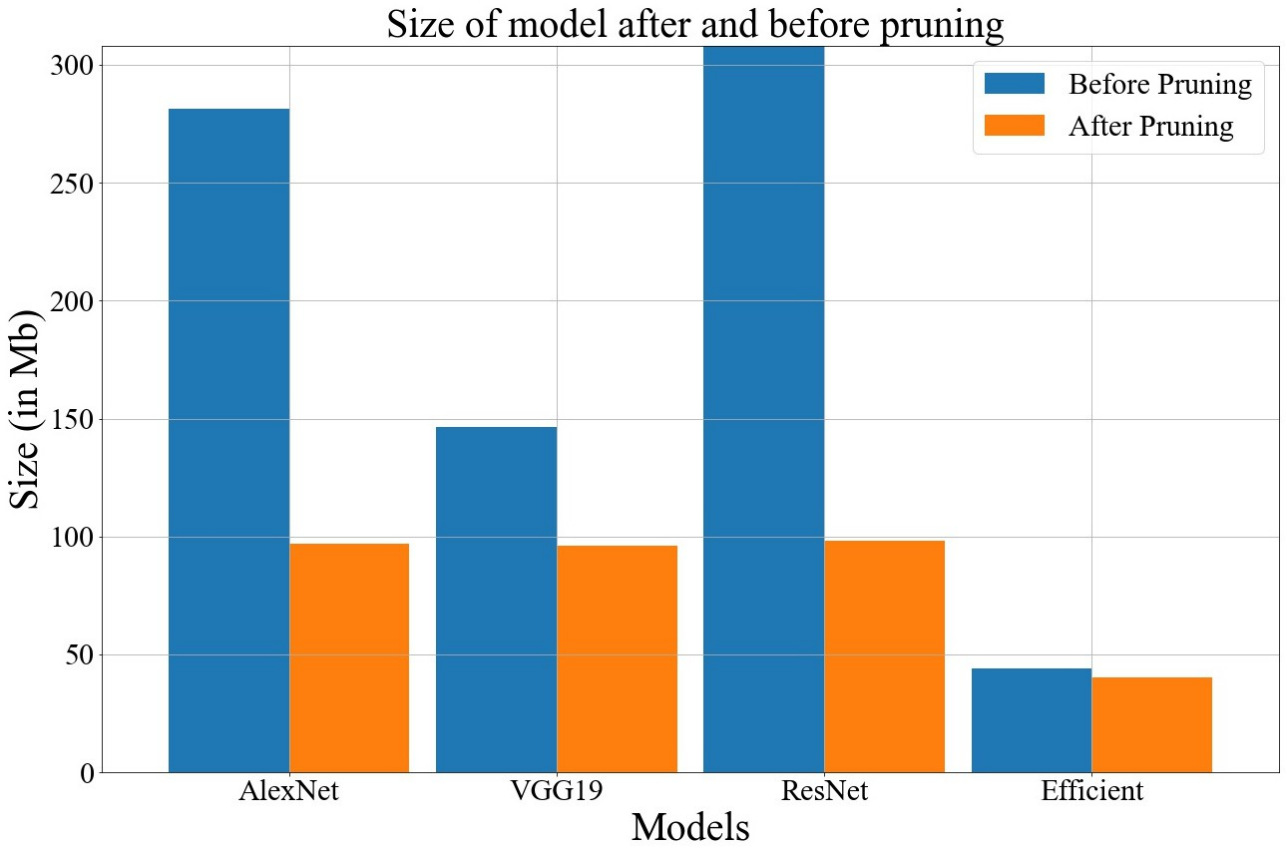
Comparison of model size after and before pruning.

**Fig 27:**
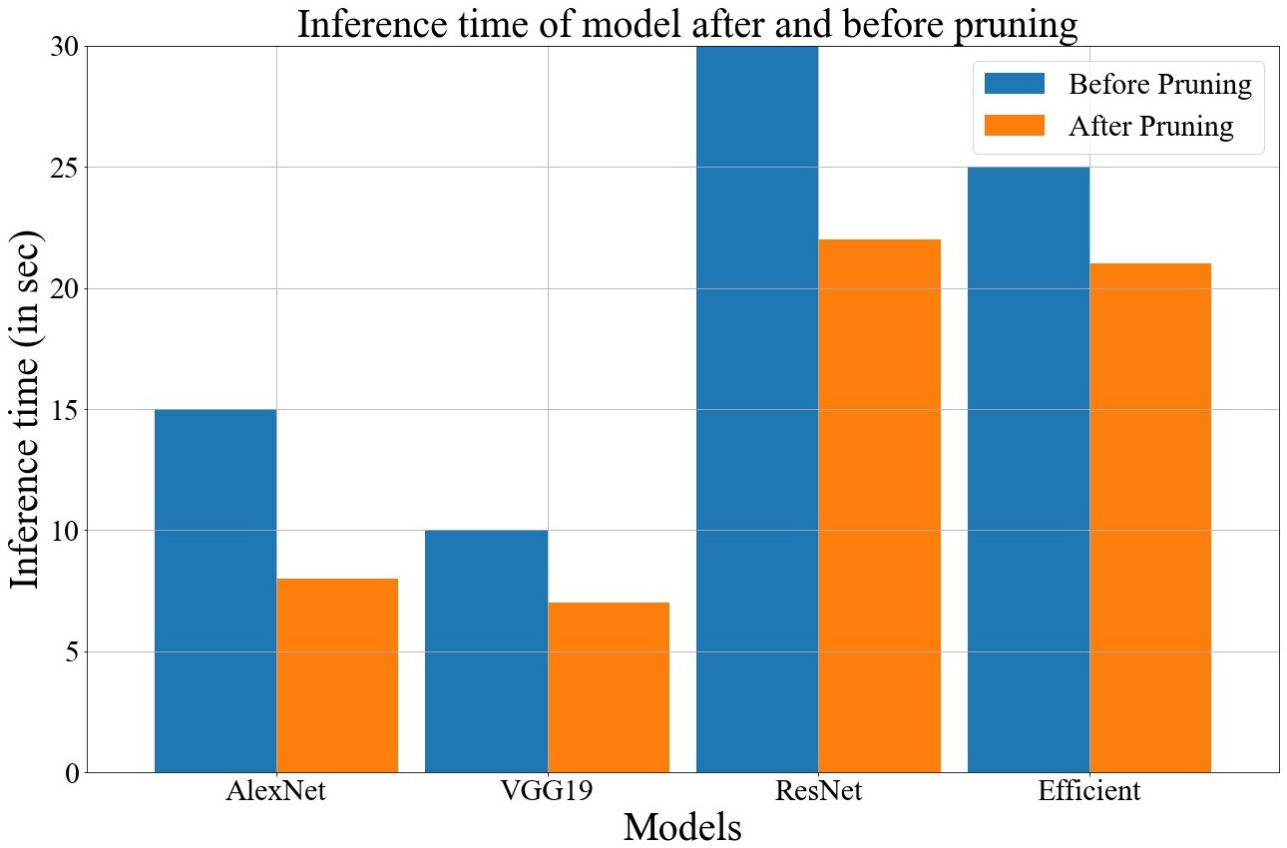
Comparison of inference time after and before pruning.

The proposed GA-based model also helps to improve inference time and storage space. The fitness function used in the method always discards hidden units or nodes that are not contributing too much to the decision-making process. The storage space and inference time before and after compression is depicted in Figs 26 and 27. Data values for Figs 26 and 27 are provided in the S1 Table (see S1 Table).

## 6 Conclusion

In the proposed work, a novel genetic algorithm-based method has been discussed to develop an optimized base model for FL. Therefore, the model can be easily deployed on such devices that are constrained by limited resources, i.e., computational power, memory, etc. For a better understanding of the proposed algorithm, all the intermediate steps of GA have been discussed with suitable examples. Here, we have developed a novel fitness function that is based on average loss, accuracy, and minimization of hidden units or nodes in the base architecture. Moreover, the strength of the chromosomes is measured using the fitness function. We have used four different deep learning architectures as the base model in FL and generated the global model by the global averaging method with an optimized base structure. The performance of all these models is compared under various performance evaluation metrics such as accuracy, F1-score, AUC-ROC, etc. We have proposed a generalized approach that can be applied to other datasets, i.e., potato & Tomato leaf disease and Indian food, to check its validity. In the tests, it was seen that EfficientNetB3 works better as a base model than other architectures. It also got 92.34% accuracy with 9 clients on a balanced dataset using the suggested GA-based method. The proposed GA-based method also helps to improve the inference time by 20%. The work can be expanded by generating the same using a GA-based approach in place of the global average method. Since the GA does not always yield the best answer, we can achieve better outcomes by adjusting a few more hyper-parameters.

## Supporting information

S1 Table Information is available at https://tinyurl.com/S1-table. (XLSX)
